# Systems-wide effects of short-term feed deprivation in obese mice

**DOI:** 10.1038/s41598-021-85020-z

**Published:** 2021-03-11

**Authors:** Daniel Andersen, Henrik Munch Roager, Li Zhang, Janne Marie Moll, Henrik Lauritz Frandsen, Niels Banhos Danneskiold-Samsøe, Axel Kornerup Hansen, Karsten Kristiansen, Tine Rask Licht, Susanne Brix

**Affiliations:** 1 Department of Biotechnology and Biomedicine, Technical University of Denmark, 2800 Kgs. Lyngby, Denmark; 2 National Food Institute, Technical University of Denmark, 2800 Kgs. Lyngby, Denmark; 3 Laboratory of Genomics and Molecular Biomedicine, Department of Biology, University of Copenhagen, 2100 Copenhagen, Denmark; 4 Department of Veterinary and Animal Sciences, University of Copenhagen, 1871 Frederiksberg C, Denmark; 5 Institute of Metagenomics, BGI-Shenzhen, Shenzhen, 518083 China; 6 Present Address: Department of Nutrition, Exercise and Sports, University of Copenhagen, 1958 Frederiksberg C, Denmark; 7 Present Address: Beijing Institute of Genomics, Chinese Academy of Sciences, Beijing, China

**Keywords:** Immunology, Microbiology, Molecular biology, Systems biology, Endocrine system and metabolic diseases

## Abstract

While prolonged fasting induces significant metabolic changes in humans and mice, less is known about systems-wide metabolic changes in response to short-term feed deprivation, which is used in experimental animal studies prior to metabolic challenge tests. We here performed a systems biology-based investigation of connections between gut bacterial composition and function, inflammatory and metabolic parameters in the intestine, liver, visceral adipose tissue, blood and urine in high-fat fed, obese mice that were feed deprived up to 12 h. The systems-wide analysis revealed that feed deprivation linked to enhanced intestinal butyric acid production and expression of the gene encoding the pro-thermogenic uncoupling protein UCP1 in visceral adipose tissue of obese mice. *Ucp1* expression was also positively associated with *Il33* expression in ileum, colon and adipose tissue as well as with the abundance of colonic *Porphyromonadaceae*, the latter also correlating to cecal butyric acid levels. Collectively, the data highlighted presence of a multi-tiered system of inter-tissue communication involving intestinal, immune and metabolic functions which is affected by feed deprivation in obese mice, thus pointing to careful use of short-feed deprivation in metabolic studies using obese mice.

## Introduction

Control of energy balance is of paramount importance for human health as long-term energy surplus leads to metabolic, inflammatory, and cardiovascular complications^1^. A number of weight regulation programs based on reduced caloric intake has been implemented such as intermittent fasting and caloric restriction strategies that may ameliorate metabolic disorders by reprogramming obesity-induced metabolic and inflammatory response patterns^2–4^.

Much mechanistic insight into molecular and immunological changes associated with obesity has been obtained from studies using the mouse as a model. In mice, extended fasting or diets that mimic fasting has been shown to profoundly alter a wide range of metabolic and physiological parameters^2,5^. By contrast, intake of an energy-dense obesogenic diet and obesity development also modulate several aspects of host homeostasis including lipid and glucose metabolism, and tissue and systemic inflammation^5,6^ associated with marked shifts in the gut microbiota^7,8^. Short-term feed deprivation in mice also reduces the influx of inflammatory monocytes in the peritoneal cavity, lung, spleen, liver, and adipose tissue^6^. Therefore, even short-term feed deprivation in obese mice may induce a metabolic shift away from the fed state. Importantly, short-term feed deprivation is often used in experimental diet-induced obesity models prior to glucose or insulin tolerance tests. Such tests most often involve removal of feed at the beginning of the light period, where feed intake is considered very low. However, in mice fed a high-fat diet for prolonged periods, a disruption of the normal feed intake pattern is observed with a continued substantial feed intake also during the light period^9,10^. Thus, particularly in relation to studies using high-fat diet-induced obese mouse models, information on how feed deprivation affects the resultant data is important, but is still lacking.

This study aimed at examining systems-wide effects of short-term feed deprivation in high-fat diet-induced obese mice. In order to obtain a systems-wide view of interlinked changes in the metabolic, inflammatory and gut microbiota profiles, we performed a comprehensive analysis of three types of high-dimensional data including intestinal bacterial composition, liver lipids and urinary metabolites in fed and feed-deprived high-fat diet-induced obese mice, and integrated these with data of cecal and fecal production of short-chain (SCFA) and branched chain fatty acids (BCFA), a priori selected gene expression profiles from the intestine (ileum and colon), liver, and visceral adipose tissue as well as plasma cytokines. We identified a feed deprivation-dependent immunometabolic circuit across multiple tissues characterized by higher levels of cecal butyric acid linked to ileal *Porphyromonadaceae* abundances and *Ucp1* expression in visceral adipose tissue, which coupled to expression of *Il33* in ileal, colonic and adipose tissue. Taken together, these findings suggest call for cautions in interpreting and comparing data obtained by experiments combining short-term feed deprivation with metabolic challenges when using high-fat diet-induced obese mice as the model system.

## Methods

### Animal experiments

Twenty male C57BL/6NTac mice (Taconic, Lille Skensved, Denmark) aged four weeks at arrival were *ad libitum* fed a standard rodent diet Altromin 1324 (Altromin, Lage, Germany) at Week 0. From Week 1, all mice were fed a synthetic D12492 high-fat diet containing 60% of the energy from fat (54.4% from lard and 5.6% from soybean oil) (Research Diets, New Brunswick, USA) for 22 weeks. Details on the diet composition is available in Zhang et al., 2017^11^. All mice were caged two-by-two with one mouse intended for feed deprivation (n = 10) and the other for *ad libitum* feeding (n = 10) in each cage. Temperature at 20–24 °C, humidity 55% ± 10% with a strict 12 h light cycle (from 6 am to 6 pm). Cages were polycarbonate Eurostandard Type III (Tecniplast, Varese, Italy), bedding was Tapvei aspen chips, and the mice had disposable Smart Home shelters, Mini Fun Tunnels, Enviro-Dri and Nestlet nesting material and Tapvei aspen size S gnawing blocks (all items purchased from Brogaarden, Lynge Denmark). One mouse died during cheek blood sampling at week 9, giving a total of n = 9 feed deprived mice. The mice used for this study originate from the study of Zhang et al., 2017^11^, but all the analyses were performed specifically for the present study, and were not included in the previous paper of Zhang et al. Moreover, the gliadin fed mice were not part of this study. The animal experiments were approved by the Danish Animal Experiments Inspectorate (Permission Number 2012-15-2934-00256\C1) and carried out in compliance with ARRIVE guidelines and Danish guidelines for experimental animal welfare.

### Sampling

Feed deprivation was initiated at the onset of the light cycle (6 am), where each of the mice were transferred to a clean cage kept in the same room in the animal facility. After 8 h and 16 min, mice were sacrificed continuously resulting in a feed deprivation span between 8 h and 16 min to 11 h and 32 min. The other mice were fed diet *ad libitum* and sacrificed based on a similar time scheme (two-by-two) on consecutive days. Details of sampling of urine, feces, intestinal lumen content, liver, eWAT and intestine is available in Zhang et al., 2017^11^.

### Biochemical measurements of plasma markers

Plasma alanine aminotransferase was measured with an ELISA kit (MyBioSource, San Diego, CA, USA). Plasma cytokines, IL-1β, IL-6, IFNγ, TNFα and IL-10, were measured using a custom V-PLEX Mouse Biomarkers ELISA kit (Meso Scale Discovery, Rockville, MD, USA).

### Intestinal SCFA and liver lipid analysis

SCFAs were analyzed in cecal and fecal samples essentially as previously described^12,13^. Liver lipids were extracted and analyzed as previously described in^14^ by Gas Chromatography Mass Spectrometry. Details are available in the supplementary material.

### Urine metabolome profiling with ultra performance liquid chromatography mass spectrometry (UPLC-MS)

Details of urine metabolite extraction, analysis and metabolite features are available in Zhang et al., 2017^11^. In short, 2 μL of diluted urine (1:100 in water) was analyzed in both negative and positive mode by a UPLC-QTOF-MS system consisting of Dionex Ultimate 3000 RS liquid chromatograph (Thermo Scientific, Sunnyvale, CA, USA) coupled to a Bruker maXis time of flight mass spectrometer equipped with an electrospray interphase (Bruker Daltonics, Bremen, Germany). The analytes were separated on a Poroshell 120 SB-C18 column with a dimension of 2.1 × 100 mm and 2.7 μm particle size (Agilent Technologies, CA, USA) based on previously reported settings^15^. The raw LC–MS data were converted to mzXML files using Bruker Compass DataAnalysis 4.2 software (Bruker Daltonics) and were then pre-processed through noise filtering, peak detection, and alignment using the open-source R package XCMS (v1.38.0)^16^. Metabolite candidates were identified by searching the accurate masses of parent ions and fragments (from MS/MS), against the METLIN^17^ and HMDB databases^18^.

### 16S rRNA gene sequencing and analysis

Details of the methods used for bacterial DNA extraction, 16S rRNA gene amplicon preparation and sequencing are available in Zhang et al., 2017^11^. Following sequencing, reads were demultiplexed, trimmed and filtered, and OTUs were generated de novo and taxonomy was assigned as in^11^. OTUs and bacterial groups at genus/family level that were less abundant than 0.02% of average numbers of total bacteria in the fed and feed deprived mice and presented in less than 50% of samples in both groups were filtered out. Microbiota α-diversity was calculated using the R package vegan (v2.5-3)^19^.

### In silico analysis of butyrate production capacity

Potential for butyrate production was identified by downloading NCBI taxIDs for all families represented in the list of statistically significant OTUs (January 12 2018). Bacterial sequences for phosphate butyryltransferase (23 September 2014), butyrate kinase (8 September 2014), 4-hydroxybutyrate CoA transferase (24 September 2014), and butyryl-CoA:acetate CoA-transferase (14 January 2014) were downloaded from Uniprot, manually curated, and used as blastp (v. 2.6.0+) queries against NCBI’s database of non-redundant proteins (nr, release from February 2 2017). Hits were identified using a fivefold cross-validated cutoff for percent similarity. Species were classified as positive for the kinase pathway if they contained hits for phosphate butyryltransferase and butyrate kinase, and positive for the transferase pathway if they contained hits for butyryl-CoA:acetate CoA-transferase. Hits for 4-hydroxybutyrate-CoA transferase were used as controls for false positive matches for butyryl-CoA:acetate CoA-transferase-hits.

### Gene expression analysis by RT-qPCR

RNA extraction, cDNA synthesis and gene expression quantification using RT-qPCR were performed as in^11^, except for inclusion of a few extra genes: *Cd36*, *Cpt1a*, *Dgat1*, *Gpr109a*, *Gpr41*, *Gpr43*, *Il18*, *Il33*, *Pyy* and *Vip* for ileum and colon, *Ppara*, *Gpr109a*, *Fgf21* in liver, and *Prdm16*, *Cidea*, *Ppargc1a*, *Il33* and *Gpr109a* for eWAT. Gene expression levels of the additional genes were quantified by RT-qPCR of the cDNA using a TaqMan Fast Universal PCR Master Mix (Applied Biosystems, Foster city, CA, USA) and a 7900HT Fast Real-time PCR system (Applied Biosystems) using validated, predesigned primers and probes (IDT, Leuven, Belgium) as specified in Table S1. Gene expression was calculated as the expression relative to the geometric mean of the expression levels of *B2m* and *Gapdh*, where mean expression levels of fed mice were set at 1.

### Statistical analysis

Unless specified, two-sided Student’s t tests (if normally distributed) or Mann–Whitney tests (if non-continuous data or not normally distributed) were performed using GraphPad Prism 6.02. Maximally one outlier from each group as detected by the Grubbs’ test (http://www.graphpad.com/quickcalcs/Grubbs1.cfm, alpha = 0.05) was excluded before the statistical tests. All p-values were adjusted for multiple testing using the Benjamini–Hochberg false discovery rate (FDR), and FDR values less than 0.05 were considered statistically significant.

Spearman’s rank correlation was used for the calculation of correlation coefficients. Networks based on Spearman correlations were built using Cytoscape v3.3.0.

Co-abundance clustering was performed using R v. 3.4.1^20^ and the R package WGCNA^21^. Signed weighted co-abundance correlations were obtained by bi-weight mid-correlations of co-abundant metabolites (log-transformed), the relative abundance of bacterial OTUs and the relative concentration of the identified liver lipids from the phospholipid and triglyceride fractions. Scale free topology criteria, β, were set as follows: β = 8 for metabolites, β = 6 for lipids, β = 14 for ileum, β = 7 for cecum, β = 9 for colon. To detect clusters using the dynamic hybrid tree-cutting algorithm, minimum cluster sizes were set at 5 (except for the lipids, which were at 3) using a deepSplit of 2 for OTUs and 4 for both metabolites and lipids.

### Ethics declarations

Animal experiments were approved by the Danish Animal Experiments Inspectorate and carried out in accordance with existing Danish guidelines for experimental animal welfare.

## Results

### Short-term feed deprivation induces distinct inter-tissue changes in obese mice

**Figure 1:**
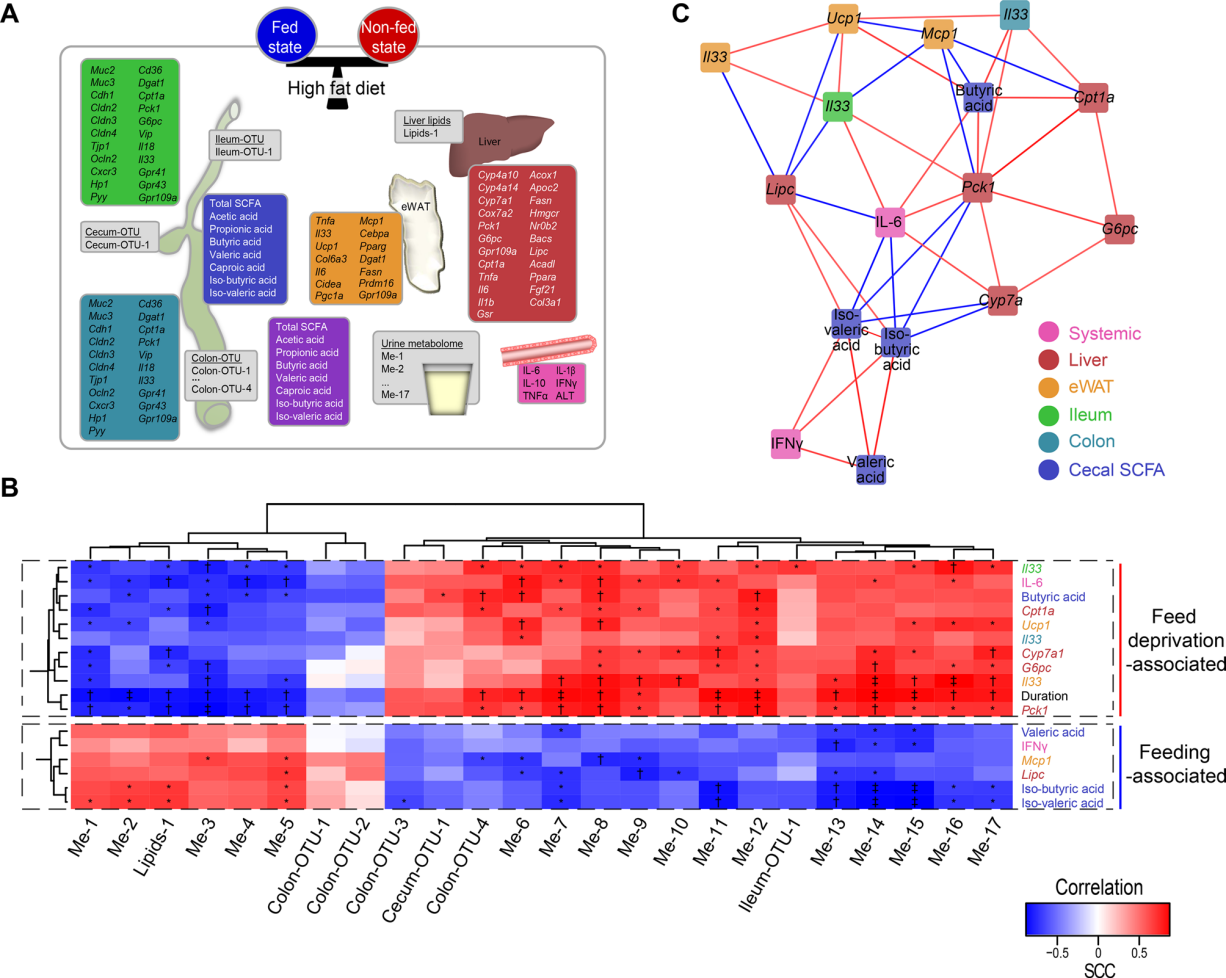
Integration of host parameters and bacterial fermentation products with feed deprivation-induced gut microbiota, liver lipids, and urine metabolite clusters. (**A**) Metabolic and inflammatory host response parameters, gut microbiota, and intestinal fermentation products determined in fed (n = 10) and feed deprived (n = 9) high-fat diet-induced obese mice. The coloring of boxes relates to measured specific factors: gene expression in liver (dark red), epididymal white adipose tissue (eWAT, orange), ileum (green) and colon (turquoise), plasma-derived cytokines and alanine aminotransferase (ALT) (pink), short-chain fatty acids from cecum (blue) and feces (purple). Feed deprivation-associated lipid species cluster, which is based on determined liver triglyceride and phospholipid species (Lipids-1), the 17 urine metabolite clusters (Me-1 to Me-17) and OTU clusters from the ileal, cecal and colonic compartments (Ileum-OTU-1, Cecum-OTU-1, Colon-OTU-1 through Colon-OTU-4) are shown in grey boxes. (**B**) Correlation heat map of the hierarchically clustered z-score-normalized low complexity parameters (y-axis) against the computed eigenvectors of lipid, metabolite and OTU clusters (x-axis) associated with feed deprivation. The duration of feed deprivation is included as a host parameter (span between 8 h and 16 min to 11 h and 32 min). The heat map color represents Spearman rank correlation coefficients (SCC), as visualized in the insert, and asterisks mark FDR-adjusted significant correlations (*, FDR < 0.05; †, FDR < 0.01; ‡, FDR < 0.001). (**C**) Network showing the internal relationship among host parameters associated with feed deprivation for edges with significant SCC (red: SCC > 0.6, blue: rho <  − 0.6, FDR < 0.05). Node colors indicate the tissue origin of the individual variable, as indicated in the legend.

To provide a systems-wide overview of effects of short-term feed deprivation on intestinal, inflammatory and metabolically regulated factors in obese mice, we examined multiple tissue and systemic parameters in C57BL/6 J mice fed an obesogenic high-fat diet for 22 weeks (Fig. 1A, colored boxes), followed by a systems biological based data integration. To examine factors linked to feed deprivation, the group of feed deprived mice were left without food between 8 and 12 h before study termination, while the remaining mice had *ad libitum* access to feed throughout the study period (fed mice). The type of feed deprivation was matched to procedures often reported in experimental animals that undergo metabolic challenge tests, where animals are deprived from food from early morning. Although mice normally are nocturnal animals, high-fat feeding off-sets the normal patterns of feed intake resulting in a continued high intake of feed during the light period^9,10^. As expected, the short-term feed deprivation resulted in no differences in whole body weight, weight of the epididymal adipose tissue (eWAT) and the liver between feed deprived and fed animals, while the regulation of the liver markers *Pck1*, *Cpt1a* and *Lipc* confirmed the feed deprived state (Fig. S1). We first performed a dimensionality reduction of the urine metabolites, OTUs and lipidomics data, resulting in varying number of clusters dependent on the data set (Fig. S2), and then integrated these clusters with the remaining data. Co-correlation analyses between all the measured metabolic and inflammatory markers (Fig. S3) independently of the fed state showed 16 out of the 102 measured tissue-specific markers to correlate to urine metabolites, OTUs and lipid clusters, of which 10 associated with the duration of feed deprivation (Fig. 1B, Fig. S4). When performing a network analysis including the above 16 tissue-specific markers, 10 markers that all associated to duration of feed deprivation (eWAT *Ucp1* and *Il33*, ileum *Il33*, colon *Il33*, liver *Pck1*, *G6pc*, *Cpt1a* and *Cyp7a1* mRNAs, systemic IL-6, and cecal butyric acid, Fig. 1B) showed a positive inter-correlation subnetwork (Fig. 1C). This subnetwork associated negatively with the feeding-associated expression of *Mcp1* mRNA in eWAT (Fig. 1C). Six markers co-clustering in the fed-associated group were represented in a second positively correlated subnetwork (liver *Lipc* mRNA, systemic Interferon-γ (IFNγ), and cecal valeric acid, iso-butyric acid and iso-valeric acid) (Fig. 1C and Fig. S5, S6). Several of these markers were negatively correlated to the markers up-regulated during feed deprivation. From the network analysis, it also appeared that *Ucp1* expression in eWAT correlated positively with expression of *Il33* in eWAT, ileum and colon*,* and cecal butyric acid levels (Fig. 1C). Also cecal butyric acid levels correlated positively with expression of *Il33* in colon, *Cpt1a* and *Pck1* in the liver*,* and blood IL-6, but not with ileal *Il33* expression. The same markers showed the strongest correlations to the eigengenes of the clusters of urinary metabolites, intestinal bacteria, and liver lipids identified to associate with feed deprivation (grey boxes of Fig. 1A) (Fig. 1B; Fig. S4 for overall correlation matrix). Some urine metabolites, OTU and lipid clusters ((Me-1 to Me-5 and Lipids-1) also correlated positively with parameters associated with the fed state (cecal valeric, iso-butyric and iso-valeric acid, blood IFN-γ, eWAT *Mcp1* and liver *Lipc* expression). Conversely, a majority of clusters (Cecum-OTU-1, Colon-OTU-3 and Colon-OTU-4, Ileum-OTU-1, and Me-6 to Me-17) were found to correlate positively with parameters associated with feed deprivation, including blood IL-6 and cecal butyric acid, and expression of *Il33* in ileum and colon, of *Ucp1* and *Il33* in eWAT, and of *Cpt1a*, *Cyp7a1*, *G6pc* and *Pck1* in the liver. Altogether, these associations suggest an interwoven connection between specific feeding state-associated host parameters across tissues.

### Feed deprivation-induced shifts in cecal butyric acid levels correlate with changes in the abundance of specific gut bacteria and *Ucp1* expression in visceral fat

Production of SCFA and BCFA by intestinal bacteria constitutes a rapid and important gut-to-host signaling mechanism, reflecting nutrient availability and activity of the gut microbiota^22^. Currently it has not been shown if short-term feed deprivation may influence intestinal production of SCFA and BCFA. We found cecal butyric acid levels to be increased in feed deprived mice while, cecal levels of the BCFAs valeric, iso-butyric and iso–valeric acid were highest in fed mice (Fig. S3A). Colonic levels of SCFA and BCFA were unaffected by the feeding state (Fig. S3B).

**Figure 2:**
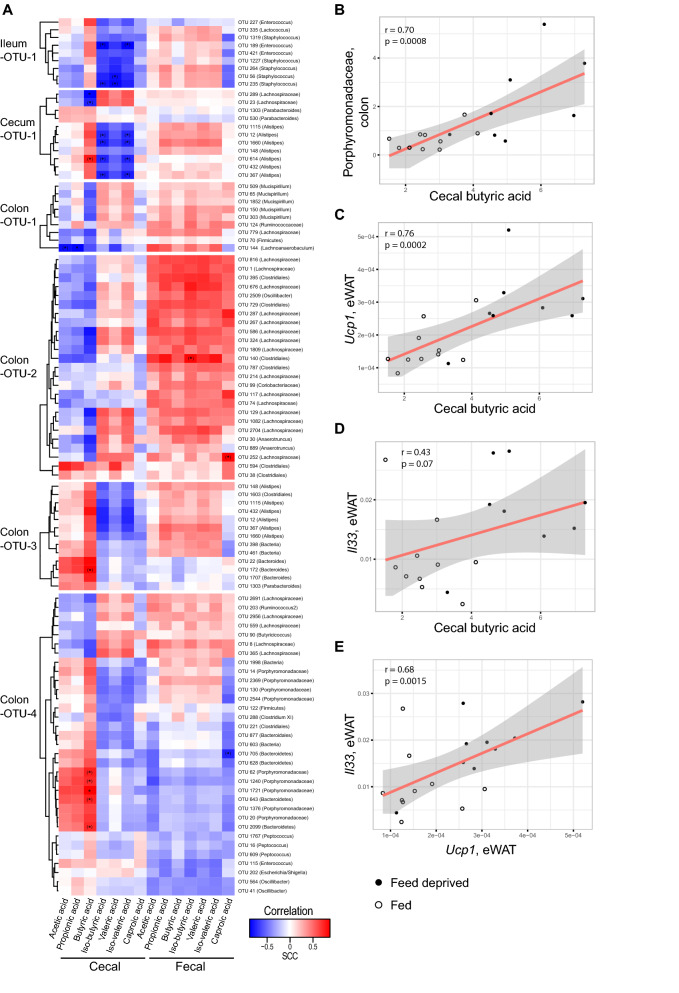
Feed deprivation-induced cecal butyric acid production links to colonic *Porphyromonadaceae* and adipose tissue expression of *Ucp1*. (**A**) Correlation heat map of all OTUs present in the clusters (Ileum-OTU-1, Cecum-OTU-1, Colon-OTU-1 to -4) associated to feed deprivation and measured levels of SCFA and BCFA in cecum and feces. OTUs were hierarchically clustered within each cluster. The heat map color represents Spearman’s rho-values (red, positive rho-values; blue, negative rho-values). Asterisks mark significant correlations (Spearman; *, FDR < 0.05; (*), FDR < 0.1). (**B**–**E**) Selected correlations related to the cecal butyric acid phenotype. Correlations of butyric acid (μg/mol) and the sum of the relative abundance of all *Porphyromonadaceae* belonging to Colon-OTU-1 to -4 (**B**), butyric acid (μg/mol) and eWAT *Ucp1* (relative expression) (**C**), butyric acid (μg/mol) and eWAT *Il33* (relative expression) (**D**), and eWAT *Ucp1* (relative expression) and eWAT *Il33* (relative expression) (**E**). The red line indicates the parametric linear regression line; grey area is the 95% confidence interval (fed mice, open circles; feed deprived mice, closed circles).

We next examined correlations between cecal SCFA and BCFA levels and the individual OTUs present in the OTU clusters associated with feed deprivation (Ileum-OTU-1, Cecum-OTU-1, Colon-OTU-1 to -4 clusters) (Fig. 2A, Table S2A). This approach identified OTUs belonging to the family *Porphyromonadaceae* to correlate positively with cecal butyric acid (Fig. 2A,B, Spearman correlation, rho = 0.70, *p* = 0.0008), while negative correlations were identified for two cecal OTUs belonging to the family *Lachnospiraceae* (Spearman's correlation, FDR < 0.1). We also evaluated by in silico analysis the genomic potential in bacteria for production of butyric acid. The in silico analysis was based on available NCBI taxIDs for bacterial families present in any of the six OTU clusters associating with feed deprivation (Fig. S3B–D), and involved identification of the presence of the butyrate kinase (*Buk*) or butyryl-CoA transferase (*But*) genes, since the presence of either of these terminal genes in the butyric acid synthesis pathways is required for bacterial butyric acid production. The in silico analysis revealed that *Porphyromonadaceae*, for which we found a positive correlation to measured butyric acid levels, and also the other families present among the OTU clusters associated with feed deprivation, contain taxIDs that have the potential for butyric acid production (Table S3). In relation to BCFA, the abundance of four cecal *Alistipes* OTUs in Cecum-OTU-1 (OTU 12, 1660, 614, and 367) was negatively correlated with cecal iso-valeric and iso-butyric acid levels (Fig. 2A). Altogether this suggests that intestinal changes in SCFA and BCFA levels during short-term feed deprivation link to changes in abundances of specific gut microbiota species. There were also apparent differences between the fed and feed deprived state in relation to bacterial abundances at the family and genus level (Fig. S7).

We next examined the correlations between intestinal butyric acid levels and visceral fat *UCP1* expression, as adipose tissues express receptors for SCFAs^23^, and butyric acid supplementation has been associated with the induction of thermogenesis in brown adipose tissue including increased PPARGC1A expression^24^. Here, we found cecal butyric acid levels to associate with *Ucp1* mRNA expression levels in eWAT (Fig. 2C). The type 2 cytokine IL-33 has previously been shown to be involved in induction of adipose tissue thermogenesis via an innate lymphoid cell type 2 (ILC2) dependent mechanism^25^. Although cecal butyric acid was not statistically significantly correlated to eWAT *Il33* mRNA expression (Fig. 2D), *Ucp1* and *Il33* expression in eWAT were found to be positively correlated (Fig. 2E). Of note, *Il33* expression displayed a remarkable synchronicity across multiple tissues as eWAT, ileal and colonic *Il33* mRNA levels were all increased in feed deprived mice and all correlated positively with eWAT *Ucp1* expression (Fig. 1C, Fig. S5). Apart from expression of *Il33*, *Ucp1* and *Mcp1*, mRNA encoding the transcription factor CEBPA*,* required for white adipocyte differentiation but not for brown-like differentiation^26^, was expressed at lower levels in feed deprived mice, but did not correlate with duration of feed deprivation (Fig. S3H). The expression of other lipo- or adipogenic genes was not different between fed and feed deprived mice (Fig. S3H).

### Network analysis identifies systems level parameters interlinked with metabolic changes in short-term feed deprived obese mice

**Figure 3:**
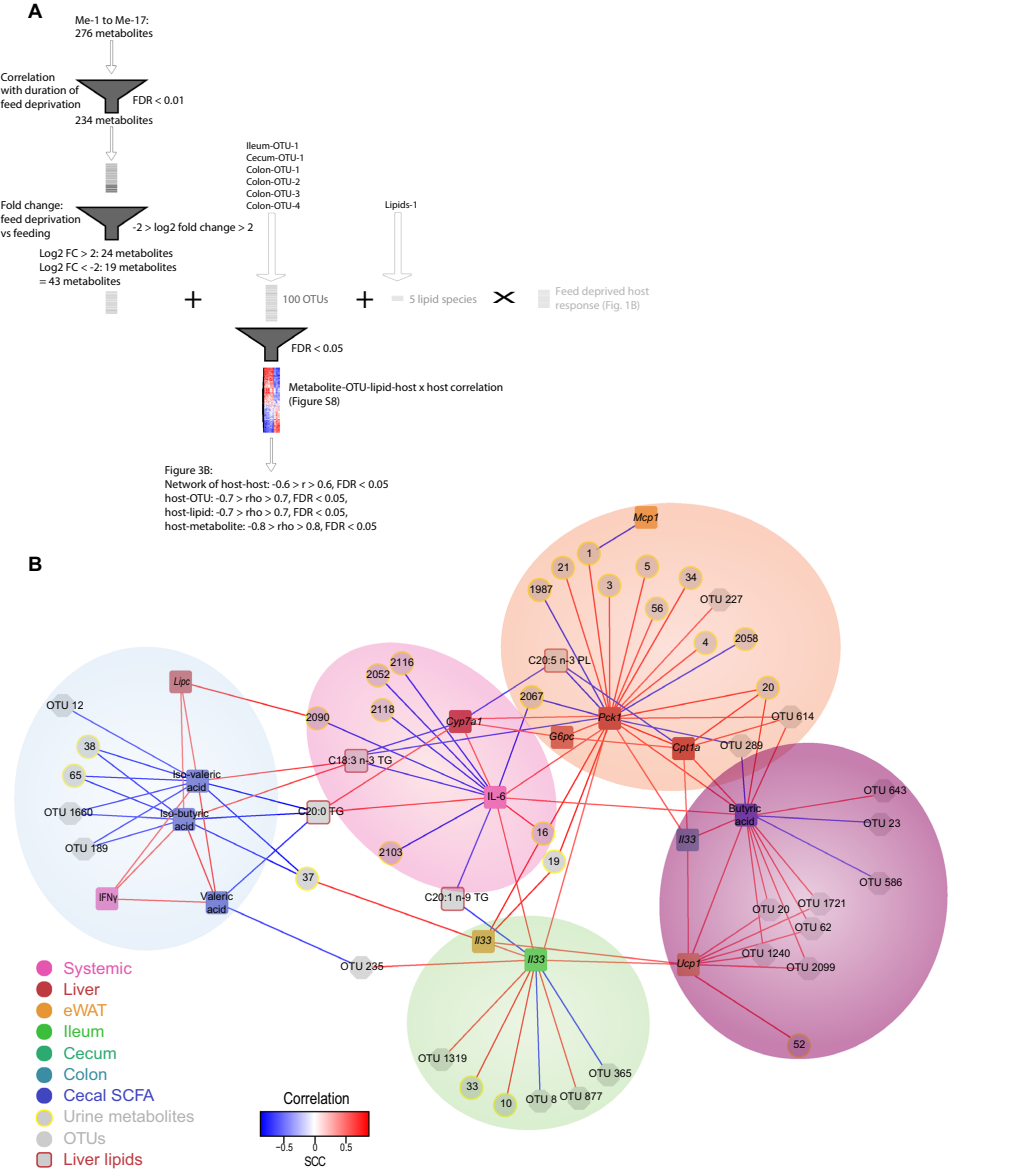
Combined network and multivariate correlation analysis to interlink feed deprivation-induced whole body parameters at the molecular level. (**A**) Step-wise approach to integrate the urine metabolome with the bacterial OTUs and liver lipids at the molecular level. All the 276 metabolites belonging to either of the 17 metabolite clusters (Me-1 to Me-17) were reduced by correlation with duration of feed deprivation (Spearman’s correlation, FDR < 0.01). The derived 234 metabolite features were similarly reduced by exclusion of metabolite features with log2 fold change < 2 and >  − 2. The resulting 43 metabolites, all 100 OTUs belonging to any of the six feed deprivation-associated bacterial OTU clusters (Ileum-OTU-1, Cecum-OTU-1, and Colon-OTU-1 to -4) and the five liver-derived lipids present in the Lipids-1 cluster were subsequently correlated with the host response parameters associated with feed deprivation (Spearman’s correlation, FDR < 0.05). (**B**) Network analysis representing the resulting significant correlations (FDR < 0.05) from (**A**). Only host-metabolite correlations with rho > 0.8 or rho <  − 0.8, host-OTU and host-lipid with rho > 0.7 or rho <  − 0.7, and host-host correlations with rho > 0.6 or rho <  − 0.6 are shown. The line color between nodes defines the correlational direction (red lines, positive correlations; blue lines, negative correlations). Identifiers for the 43 metabolites, 100 OTUs and 5 lipids are shown in Table S2A-C. The resultant five modules are shaded in individual colors (blue, green, orange, pink, purple). Node colors and shapes are representative of the data type.

To identify inter-tissue links between individual metabolic and inflammatory factors within the intestine, liver, blood, adipose tissue, and urine, we next conducted a network analysis aiming to integrate all feed deprivation-associated bacterial and host-related factors at the molecular level (Fig. 3A). Data integration was performed in a structured manner, by first identifying feed deprived-associated metabolites that correlated with changes in the intestinal bacteria composition, liver lipids or the main host factor phenotype, thus being potential candidates as systemic mediators of the bacterial changes or byproducts of the microbiota-associated changes in host metabolism induced by feed deprivation. In order to reduce the complexity of the urine metabolites, the 276 metabolite features with retention > 35 s present in the duration of feed deprivation-associated clusters (Me-1 to Me-17) were removed if they did not correlate to duration of feed deprivation (FDR < 0.01) (Fig. 3A). The remaining 234 metabolites were subsequently filtered by only including metabolites that showed at least an average twofold change between fed and feed deprived mice, resulting in 43 metabolites all presented with considerable differences in levels between the two feeding states (Table S2B). Several of these metabolites could not be identified using either the METLIN or HMDB databases when searching for m/z identification. Some metabolites differed due to reduced nutrient intake of the feed deprived mice and were consequently more abundant among fed mice (2004, 2043, 2062, 2066, 2068, 2090, 2088, 2089, 2093, 2098, 2103, 2113, 2116 and 2118, annotated either as disaccharides, dipeptides or isotopes hereof, or alternatively as O-glycosyl compound fragments). Surprisingly, metabolite 2058 identified as hydroxybutyrylcarnitine, a known ketosis-induced metabolite, was increased in fed mice (Table S2B). Other metabolites were clearly a consequence of the metabolic feed deprivation-induced response of the host, such as the metabolites 10, 12, 13, 14, and 15, which were identified as cortisol derivatives.

Co-correlation analyses between the 43 metabolites and the intestinal bacterial OTUs present in the feed deprivation-associated OTU-clusters (Ileum-OTU-1, Cecum-OTU-1, Colon-OTU-1 to -4), and the five liver lipid species (Lipids-1) (Table S2C) allowed for identification of individual urine metabolites, OTUs and lipids that correlated most strongly with the observed metabolic phenotype associating with feed deprivation (Fig. 3A and Fig. S8, FDR < 0.05). As pointed out in Fig. S8, multiple urine metabolites showed a strong correlation with the feed duration-associated host factors. The interconnections between host factors, metabolites, OTUs and lipids were visualized in a network structure (Fig. 3B). Since the metabolite features exhibit dominant inter-correlations, only very strong Spearman correlation coefficients (rho > 0.8 or rho < − 0.8) between metabolites and host factors are shown, whereas the cutoff for host-OTU and host-lipid Spearman correlation coefficients are set at − 0.7 > rho > 0.7, and host-host correlations (which show a relatively lower level of association with each other), are set at − 0.6 > rho > 0.6.

The resulting network comprised five major modules (blue, green, orange, pink and purple circles) that mainly differed based on involved host factors. The fed state-associated module (light blue) consisted of systemic IFNγ, the hepatic lipase *Lipc*, and cecal valeric, iso-valeric and iso-butyric acid. The two BCFAs, iso-valeric and iso-butyric acid, in cecum were negatively associated with three urine metabolites (37, 38 and 65) that we were unable to annotate. Two *Alistipes* spp. were found to link to this module; *Alistipes* spp. (OTU 12 from colon) correlated negatively with iso-valeric acid, and *Alistipes spp*. (OTU 1660 from cecum) correlated negatively with iso-valeric and iso-butyric acid, as did *Enterococcus spp*. (OTU 189 from ileum).

The other four modules were dominated by host factors increased during feed deprivation. One of these was dominated by *Il33* mRNA from both ileum and adipose tissue (green). While adipose tissue *Il33* mRNA correlated positively with three unidentifiable urine metabolites (16, 19 and 37), ileum *Il33* mRNA correlated positively with the two urine metabolites 10 (tetrahydrocortisone isotype or dihydrocortisol) and 33 (gluconolactone, galactonolactone or gulonolactone). Ileal *Il33* mRNA also linked positively with two OTUs (OTU 877, *Bacteroidales* from colon; and OTU 1319, *Staphylococcus* from ileum), and negatively with two OTUs from colon (OTU 8 and 365, both *Lachnospiraceae*). Another nearby module (purple) was characterized by the presence of *Ucp1* mRNA (positively linked to ileum and adipose tissue *Il33* mRNA), colon *Il33* mRNA and cecal butyric acid, which all correlated positively with each other. *Ucp1* mRNA also correlated positively with the unannotated urine metabolite 52. Interestingly, five OTUs correlated positively with *Ucp1* mRNA as well as cecal butyric acid levels. All of these were assigned to the *Bacteroidetes* class, while four of these were further assigned to the *Porphyromonadaceae* family (OTU 20, 62, 1240, 1721). In addition to this, OTU 643 (*Bacteroidetes*) and OTU 614 (*Alistipes*) correlated positively to cecal butyric acid, while OTU 23 (cecum), OTU 289 (cecum) and OTU 586 (colon) (all *Lachnospiraceae*) correlated negatively with cecal butyric acid levels.

Another module (orange) consisted of multiple urine metabolites correlating with liver *Pck1* mRNA, encoding the key gluconeogenic regulator PCK1, either positively (4, 5, 16, 19, 20, 34 and 56, all unidentified; 1, Hydroxysanguinarine; 3, Hydroxysanguinarine isotope) or negatively (1987 and 2067, both unidentified; and 2058, Hydroxybutyrylcarnitine) and the two *n*-3 lipids, the phospholipid derivative C20:5 *n*-3 and the triglyceride derivative C18:3 *n*-3. Of note, C20:5 *n*-3 and C18:3 *n*-3 correlated in an inverse manner with liver gene expression of *Cyp7a1* and *Pck1*, in addition to *Cpt1a* for C20:5 *n*-3. Liver *Pck1* mRNA is a hub in the network and is involved in a positive correlation network with *Cyp7a1* (encoding the rate-limiting enzyme in bile acid synthesis), *G6pc* (encoding G6PC involved in gluconeogenesis) and *Cpt1A* (encoding CPT1a involved in mitochondrial fatty acid β-oxidation and ketogenesis), altogether highlighting the elaborate and numerous changes short-term feed deprivation induces on host metabolism.

The last module (pink) consisted of the systemic cytokine IL-6, increased in feed deprived obese mice, which correlated negatively with several metabolites (16, 2052, 2067, all unidentified, and 2090, 2103, 2116, and 2118, all dipeptides) and the triglyceride derivatives C18:3 *n*-3 and C20:1 *n*-9, and positively with the triglyceride derived C20:0 lipid.

Altogether, these data emphasize that short-term feed deprivation induces multiple systems-wide changes in high-fed diet-induced obese mice as identified by systems biological analyses.

## Discussion

The mammalian body and the gut microbiota constitute a flexible holobiont, permitting rapid adaptation to changes in nutrient availability. Despite—or perhaps because of—being a recurring phenomenon, cessation of nutrient intake imposes a significant change to multiple interrelated systems of the mammalian body. The current capability of unbiased and untargeted high-dimensional methods—such as metagenomics, lipidomics and metabolomics—allows analysis of the impact of the feeding status on these parameters. To extract key phenotypic factors associated with the feed deprivation, we here applied a robust data reduction method that enabled identification of the most radically altered factors and their individual relationships across tissues.

We identified marked changes in host metabolic regulation following short-term feed deprivation in high-fat diet-induced obese animals; a change that was defined as networks of potential biological importance between specific intestinal bacteria and their fermentation products, gene expression in ileum, liver, and visceral adipose tissue, liver lipid species, and urinary metabolites. Of note, especially the gut bacterial composition and activity were found to be markedly changed in response to feed deprivation in the obese mice with a reduction in cecal branched-chain fatty acids and an increase in butyric acid concentration coinciding with marked changes in the gut microbiota composition. The most significant alterations in bacterial composition were the higher relative abundances of *Porphyromonadaceae* in cecum and colon, an observation, which correlated robustly with cecal butyric acid concentrations. This, in combination with our identification of key genes encoding butyrate synthetic enzymes in many *Porphyromonadaceae*, suggest the involvement of intestinal *Porphyromonadaceae* spp. in production of intestinal butyrate during feed deprivation. Butyric acid is involved in the maintenance of colonic epithelial homeostasis and the regulation of intestinal inflammation^27,28^. Low abundance of butyric acid producing bacteria has been associated with increased risk for development of type 2 diabetes^29^ and SCFA supplementation studies have identified a link between SCFAs and energy expenditure associated with expression of the pro-thermogenic regulators PPARGC1A and UCP1 in brown adipose tissue^24^. Moreover, the addition of acetate, propionate or butyrate to a high-fat diet prevents diet-induced body weight gain and increases energy expenditure^30^, and induces the expression of beige adipocyte markers in epididymal adipose tissue^23^. Additionally, intraperitoneal injection of sodium butyrate in *db*/*db* mice lowers inflammation in subcutaneous and epididymal adipose tissue via the inhibition of NLRP3 activation^31^. These findings are in line with our observation that an increase in cecal butyric acid concentration even upon short-term feed deprivation was associated with the transcriptional induction of a pro-thermogenic phenotype marked by increased expression of *Ucp1* and decreased expression of the pro-inflammatory cytokine MCP1 in visceral adipose tissue.

We observed a strong correlation between eWAT *Il33* and *Ucp1* expression, which is in line with previous reports^32,33^, suggesting the involvement of IL-33 in promoting a pro-thermogenic phenotype in white adipose tissue as also observed in caloric restricted mice^34^ or obese mice on a 30 day every-other-day fasting regimen^35^. In addition to epididymal adipose tissue changes, we found short-term feed deprivation to be associated with an intestinal reprogramming mirroring the response in the adipose tissue, as concomitantly higher ileal and colonic *Il33* expressions were induced by feed deprivation. IL-33 is linked to propagation of an innate and adaptive type 2 immune response important for maintenance of a homeostatic intestinal environment^36–38^ and insulin regulation^28,39^. IL-33 is also reported to expand regulatory T cells^40,41^ and to favor a classical adaptive Th2 polarization^42^. Cleavage of IL-33 by inflammasome-activated caspases (e.g. Caspase-1) inactivates the IL-33 protein^43^. Since inflammasome activation via NLRP3 can be reduced by either butyric acid^31^ or by the ketone body beta-hydroxy-butyrate (derived from either butyric acid or ketogenic amino acids)^44^, it is possible that increased levels of these metabolites may increase IL-33 levels in the short-term feed deprived mice.

The liver of feed deprived obese mice also exhibited typical signs of metabolic shifts, characterized by altered expression of targets of the nutrient and lipid sensing master regulators Farnesoid X Receptor (FXR) (typical of the fed state) or Peroxisome Proliferator-Activated Receptor α (PPARA) (typical of the feed deprived state)^39^. Targets of these include the genes regulated by feed deprivation: *Cyp7a1*, and *G6pc* and *Pck1* (all inhibited by the FXR-induced corepressor *Nr0b2* (or Small Heterodimer Partner, SHP)) or *Cpt1a* (induced by PPARA)^45,46^. In addition, CPT1A promotes generation of acetyl-CoA, which in turn increases PEPCK/gluconeogenesis and functions as a substrate in ketogenesis^47^, further explaining the observed positive correlations.

Every other day fasting (EODF) of mice has been shown to alter the gut microbiota composition and, interestingly, mice improve their metabolic homeostasis when receiving EODF fecal microbiota transplants^35^. Insight into feed deprivation-induced changes in the abundance of *Porphyromonadaceae* is limited, however, in one study, members of the family *Porphyromonadaceae* were identified as being more abundant in life-long calorie-restricted rather than *ad libitum* high-fat fed mice^48^. Conversely, in a short-term study of two weeks caloric restriction of low fat fed mice, members of the family *Porphyromonadaceae* were less abundant in the feces of calorie-restricted compared to ad libitum fed mice^49^. In light of this, the current data suggest that the combination of high-fat feeding and short-term feed deprivation or long-term caloric restriction may cause the relative increase in *Porphyromonadaceae* abundance. Zhang et al. (2013) propose that calorie restriction limits nutrients for the gut microbiota and the host, and that the host acts to increase protein and lipid absorption, thus increasing the relative proportion of fiber in the gut and thereby substrates for butyrate production^48^.

Several urinary metabolites showed consistent correlations with the changes in metabolic and inflammatory properties in the feed deprived obese mice emphasizing that the response to short-term feed deprivation is not just limited to gene expression changes, but also manifested in changes in urine metabolite levels. In addition to various metabolites derived from cortisol (metabolites 10, 12, 13, 14 and 15), a well-characterized fasting-induced hormone stimulating gluconeogenesis, suppressing the immune system, and modifying fat and carbohydrate metabolism, several novel metabolites were identified. While the functions (if any) of most of these metabolites have been poorly characterized, their identification in urine indicates a strong influence of short-term feed deprivation of obese mice.

One strength of this study is the finding of specific and numerous changes associated with experimental short-term feed deprivation in obese mice. The identified changes in inflammatory and metabolic parameters point to a positive effect of short-term feed deprivation in obese mice involving propagation of a tissue-sustaining type 2 immune profile in intestinal tissue and visceral fat being coupled to a fat-burning phenotype suggestive of short-term improvements in inflammatory and metabolic health. This notion is in keeping with findings from other studies demonstrating that despite of slightly different regimens of intermittent feeding, and differences in the effect of housing temperature, the common denominator associated with the beneficial effects on whole body metabolism is an upregulated type 2 immune profile^50^. The intricate relationship between the multiple interwoven systems shown here is based on correlations, and thus we cannot conclude on the causal relationships between the phenotypes. Still, these data point out key interaction hubs involved in the systems-wide effects induced by short-term feed deprivation of obese mice.

Altogether, our systems-level analysis suggests a multi-tiered system of inter-tissue communication involving enhanced abundances of *Porphyromonadaceae* and butyric acid production in the intestine, coupled with increased expression of the type 2 immune regulator *Il33* and visceral adipose expression of *Ucp1* during short-term feed deprivation in obese mice. Moreover, the data highlight the type of multi-faceted factors, including *Il33* of adipose and intestinal tissues, thermogenesis-associated markers such as *Ucp1* in eWAT, *Mcp1* in eWAT, liver metabolic markers such as *Lipc*, *Cpt1a* and *Pck1* in addition to cecal butyric acid, iso-valeric, and -butyric acid, and systemic IL-6 to be considered in studies using feed deprivation in experimental obese models to standardize nutrient intake when examining metabolic function via e.g. glucose and insulin tolerance tests.

## Supplementary Information

Supplementary Figures and Tables

## Data Availability

The datasets used during the current study are available from the corresponding author on reasonable request.

## Abbreviations

BCAA: Branched-chain amino acids
BCFA: Branched chain fatty acids
eWAT: Epididymal adipose tissue
ILC2: Innate lymphoid cell type 2
SCFA: Short-chain fatty acids
UCP1: Uncoupling protein 1
