# Supplementary material for: Systems-wide effects of short-term feed deprivation in obese mice
Source: Sci Rep. 2021 Mar 11;11:5716. doi: 10.1038/s41598-021-85020-z (PMC7952545; doi:10.1038/s41598-021-85020-z)
Supplement: Supplementary file 1 — Supplementary Figures and Tables [file 41598_2021_85020_MOESM1_ESM.pdf]

SUPPLEMENTARY MATERIAL

Systems-wide effects of short-term feed deprivation in obese mice

Daniel Andersen[1], Henrik Munch Roager[2Δ], Li Zhang[2&], Janne Marie Moll[1], Henrik Lauritz Frandsen[2], Niels Banhos Danneskiold-Samsøe[3], Axel Kornerup Hansen[4], Karsten Kristiansen[3,5], Tine Rask Licht[2], Susanne Brix[1#]

[1]Department of Biotechnology and Biomedicine, Technical University of Denmark, DK-2800 Kgs. Lyngby, Denmark

[2]National Food Institute, Technical University of Denmark, DK-2800 Kgs. Lyngby, Denmark

[3]Laboratory of Genomics and Molecular Biomedicine, Department of Biology, University of Copenhagen, Universitetsparken 13, DK-2100 Copenhagen, Denmark

[4]Department of Veterinary and Animal Sciences, University of Copenhagen, 1871 Frederiksberg C, Denmark

[5]Institute of Metagenomics, BGI-Shenzhen, Shenzhen 518083, China

[#]Corresponding author: Susanne Brix, sbp@bio.dtu.dk, Department of Biotechnology and Biomedicine, Technical University of Denmark

[Δ]Current affiliation: Department of Nutrition, Exercise and Sports, University of Copenhagen, DK-1958 Frederiksberg C, Denmark

[&]Current affiliation: Beijing Institute of Genomics, Chinese Academy of Sciences, Beijing, China

Running title: Systems-wide effects of short-term feed deprivation

Abbreviations: BCAA: branched-chain amino acids, BCFA: branched chain fatty acids, eWAT: epididymal adipose tissue, ILC2: innate lymphoid cell type 2, SCFA: short-chain fatty acids, UCP1: uncoupling protein 1.

SUPPLEMENTARY METHODS

Intestinal SCFA analysis

Frozen cecum content and fecal pellets were thawed on ice. Cecum contents (5-25 mg) were homogenized in 250 µl methanol, 250 µL Milli-Q water and 10 µL internal standard (100 mM 2-ethylbutyric acid in 12% formic acid, Sigma-Aldrich, St.Louis, MO, USA) using a micro-homogenizer. Similarly, one or two fecal pellets per sample were homogenized in 1.5 mL water and 100 µl internal standard using a bead-beater, and incubated for 10 min at room-temperature with slow shaking. Acidity of samples was adjusted to pH = 2-3 using 3M HCl. The samples were then centrifuged at 10,000 g for 10 min, and supernatants were filtered through 0.45 µm Phenex-NY syringe filters (Phenomenex, Værløse, Denmark). External calibration was performed using standard solution mixtures of acetic acid, propionic acid, butyric acid, iso-butyric acid, valeric acid, iso-valeric acid, caproic acid and 2-ethylbutyric acid (Sigma-Aldrich) in the concentrations 10, 20, 50, 100, 250, 500 and 1,000 mM with extra acetic acid, propionic acid and butyric acid in the concentrations 2000 and 5000 mM. Aliquots (3 µL) of each sample were injected into a HP 6890 GC system (Agilent Technologies, Santa Clara, CA, USA) with a CP-FFA WCOT fused silica capillary column (25 m x 0.53 mm i.d. coated with 1 µm film thickness, Chrompack, EA Middelburg, The Netherlands). The carrier gas was helium at a flow rate of 20 mL/min. The initial oven temperature of 60 °C was maintained for 0.25 min, raised to 180 °C at 8 °C/min and held for 3 min, then increased to 215 °C at 20 °C/min, and finally held at 215 °C for 5 min. The temperature of the front inlet detector and the injector was 250 °C. The flow rates of hydrogen, air and helium as makeup gas were 40, 450, and 45 mL/min, respectively. The run time for each analysis was 22 min. Data handling was performed using the OpenLAB Chromatography Data System ChemStation Edition software (Rev.A.10.02). The concentration of SCFA in the samples was calculated against the individual external standards, and adjusted according to the loss of internal standard.

Liver lipid analysis

Triglycerides and phospholipids were extracted from 200 mg liver tissue samples by mixing with 1100 µL ice-cold methanol, three ceramic beads and 100 µL internal standard (C19:0), followed by homogenization using a Mini-Beadbeater-96 (Biospec) for three minutes. After transfer of the homogenate to 12 mL glass tubes, 3000 µL chloroform:methanol (2:1) was added followed by 2300 µL chloroform with 200 µg/mL 2,6-Di-tert-butyl-4-methylphenol. 1300 µL 0.73 % NaCl was added and tubes were vortexed and centrifuged (5 min., 3000 rpm, 4 ºC). The lower phase was transferred to a clean tube, and after evaporation of the solvent, lipids were dissolved in 250 µL chloroform:methanol (2:1) and stored at -20 ºC until analysis.

For fractionation, the aminopropyl cartridges (Phenomenex Strata NH2, 500mg) were washed twice with 1 mL chloroform:methanol (2:1), dried, and then washed with 2x1000 µL hexane. The lipid extracts were dried down, dissolved in 200 µL chloroform, and then added to the cartridge. After elution of cholesterol esters with 2 times 2.5 mL hexane (fraction 1), triglycerides were eluted using 2 times 2.5 ml hexane:chloroform:ethylacetate (100:5:5) (fraction 2), then cholesterol, diacylglycerols and ceramide (fraction 3) using three times 2.0 mL chloroform:methanol (23:1), free fatty acids (fraction 4) with 5 ml 2% acetic acid in diethylether, and, finally, phospholipids (fraction 5) with 5 mL methanol. Fractions were dried using nitrogen and triglycerides redissolved in 200 L heptane, and phospholipids redissolved in 400 L chloroform:methanol (2:1). To produce fatty acid methyl esters, lipids were then redissolved in 0.5 M NaOH and refluxed for five minutes at 80 °C in a block heater, and then cooled to room temperature in a water bath. Methylation was obtained using 20 % BF3 in methanol as methylation reagent and 0.1 % hydroquinone in methanol was added for antioxidative purposes. The samples were once again refluxed (two minutes at 80 °C) and cooled down to room temperature. To each sample, 0.73 % NaCl solution was added, followed by thorough vortexing. Heptane was added and the samples were vortexed and centrifuged for one minute at 4000 rpm. The heptane phase was transferred to a 3 ml methylation glass tube. Finally, heptane was added and the centrifugation and transfer process repeated. The lower phase and remaining upper phase, if any, was discarded. Saturated alkaline NaCl-solution (approximately 40 g NaCl and 150 mg K2CO3 per 100 ml) was added to the collected heptane phases, followed by vortexing and centrifugation for one minute at 4000 rpm. The resulting heptane phase was transferred to GC vials. The phospholipids and triglycerides were separated on a 60 m SP-2380 column (Sigma-Aldrich, Brøndby, DK) using a HP 6890 GC system (Agilent Technologies, Santa Clara, CA, USA) in split mode using Helium as carrier-gas. A split-ratio of 1:10 was used and 5 µL of sample was injected. The temperature started at 50 °C for three minutes, and was raised to 160 °C with 15 °C/min. From here the temperature was raised to 182 °C with 1 °C/min. Again the temperature was raised, this time by 10 °C/min until 200 °C/min was reached, which was held for 15 minutes. Finally, the temperature was raised by 30.0 °C/min until the final temperature of 225 °C was reached an then held for 12 minutes before resetting of the system to the initial temperature.

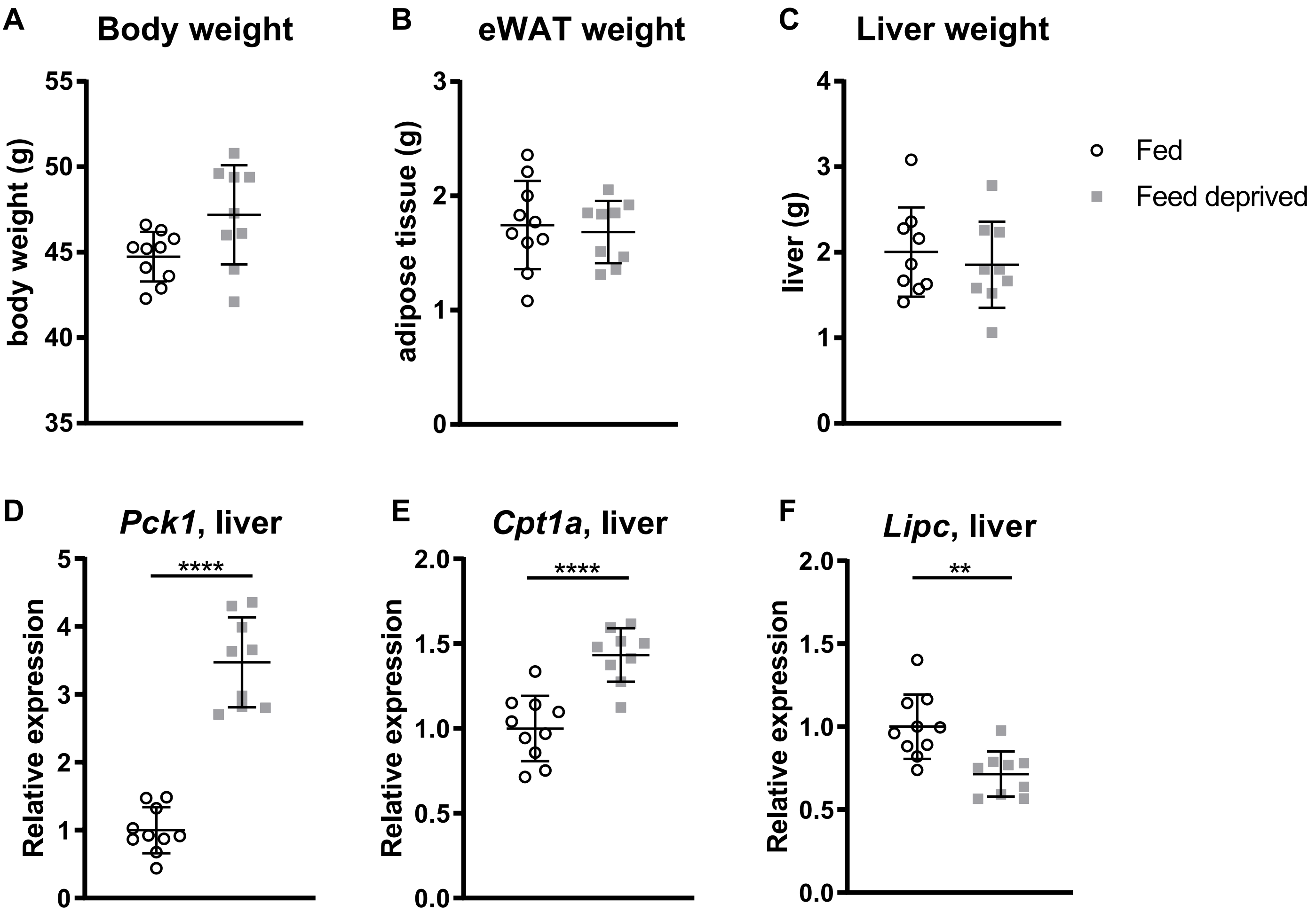

Figure S1. Body and tissue weights at study termination.
C57BL/6J mice were fed a high-fat diet for 22 weeks. Before sacrifice, nine mice were subjected to feed deprivation for 8 hours and 16 minutes to 11 hours and 32 minutes, while the remaining ten mice were provided ad libitum access to feed throughout the study period. A. Whole body weight prior to feed deprivation. B. Epididymal adipose tissue weight, and C, liver weight registered after termination. D-F. Liver markers used to define feed deprivation status, as assessed based on gene expression. Asterisks mark the statistically significant differences in the relative expression between fed and feed deprived mice (t test; **, p-value < 0.01; ****, p-value < 0.0001). Fed mice (white, open circles), n=10; feed deprived mice (grey squares), n= 9, error bars display the mean and standard deviation.

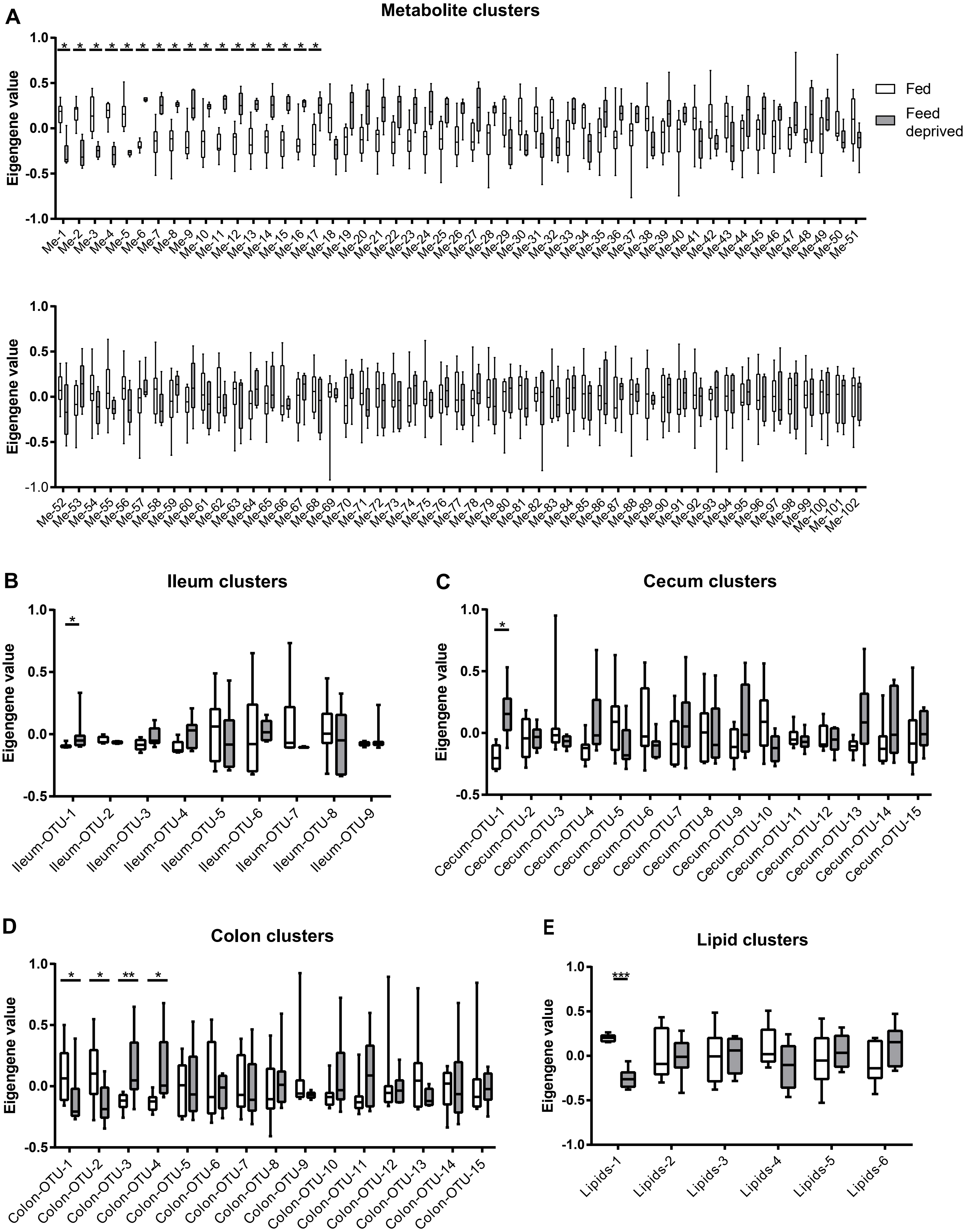

Figure S2. Eigengene values of all urine metabolite, gut microbiota and liver lipid clusters from WGCNA. Co-abundance clusters from the urine metabolome (A), ileum (B), cecum (C) and colon (D) microbiome at the OTU level, and liver lipids (E). Asterisks mark the significant differences in eigengene values between fed and feed deprived mice (t test; *, FDR < 0.05; **, FDR < 0.01). The minimum cluster size was set at five for metabolites and OTUs, and at three for the liver lipids. Boxes show the 25-75 percentile with the median shown as a line, whiskers extending from 5-95 percentile.

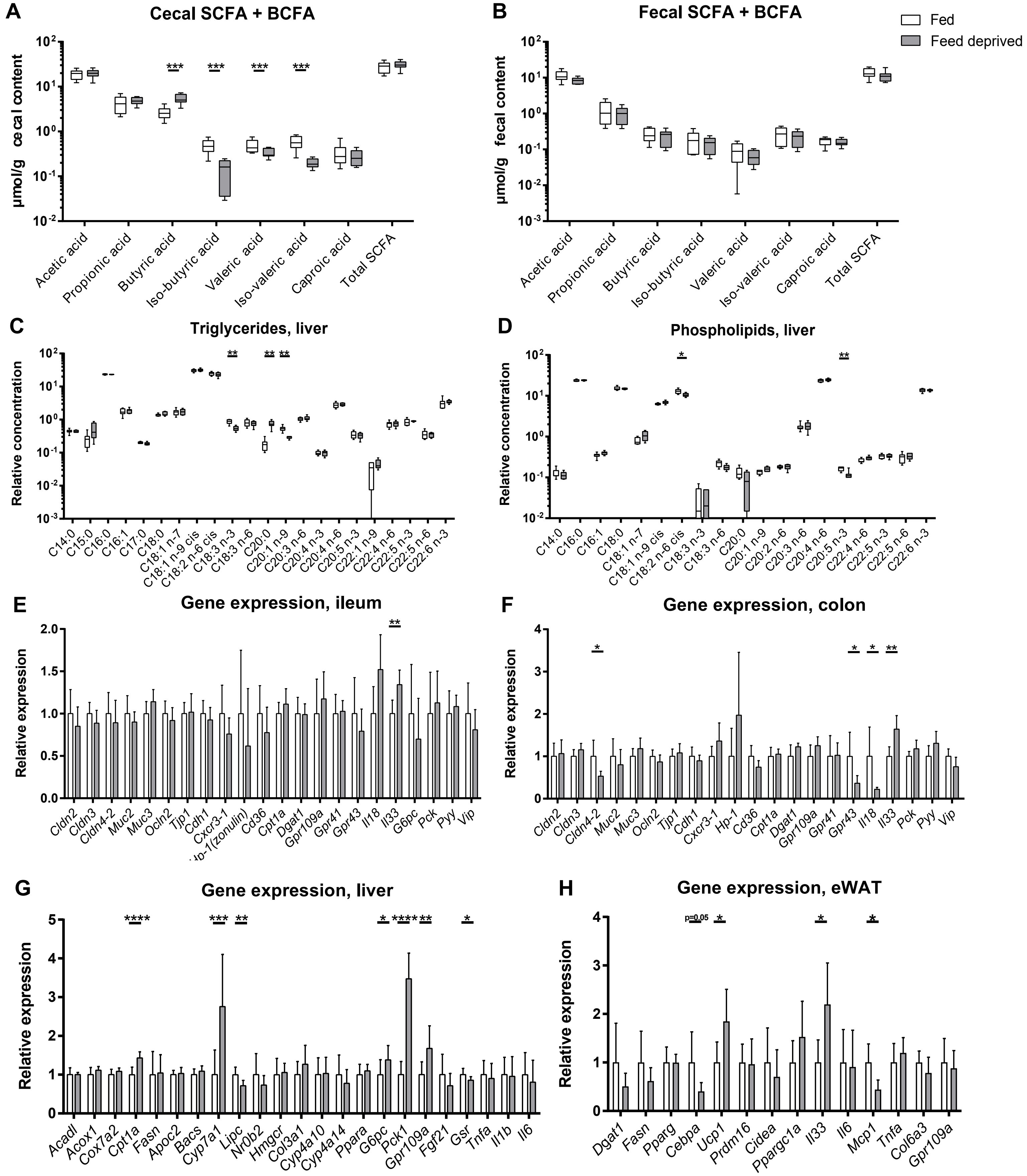

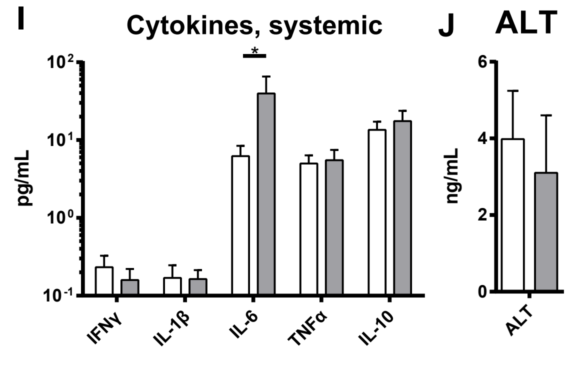

Figure S3. Univariate analysis of bacterial fermentation products, metabolic and inflammatory host factors.

Tissues from fed (n=10) and feed deprived (n=9) obese mice. A. Cecal SCFAs and BCFAs levels. B. Fecal SCFAs and BCFAs levels. C. Relative concentrations of liver lipids from the triglyceride fraction or D, the phospholipid fraction. Quantitative RT-PCR of ileum (E), colon (F), liver (G) and epididymal white adipose tissue (eWAT, H). I. Plasma protein levels of cytokines. J. Plasma alanine aminotransferase concentration. A-D, shown as boxplots of the mean with error bars marking the 5th-95th percentiles, E-I, boxplots displaying the mean with standard deviation. RT-qPCR data are shown as relative expression normalized to the fed mice. Fed mice, white boxes; feed deprived mice, grey boxes. For all statistics: t test; *, FDR < 0.05; **, FDR < 0.01; ***, FDR < 0.001; ****, FDR < 0.0001.

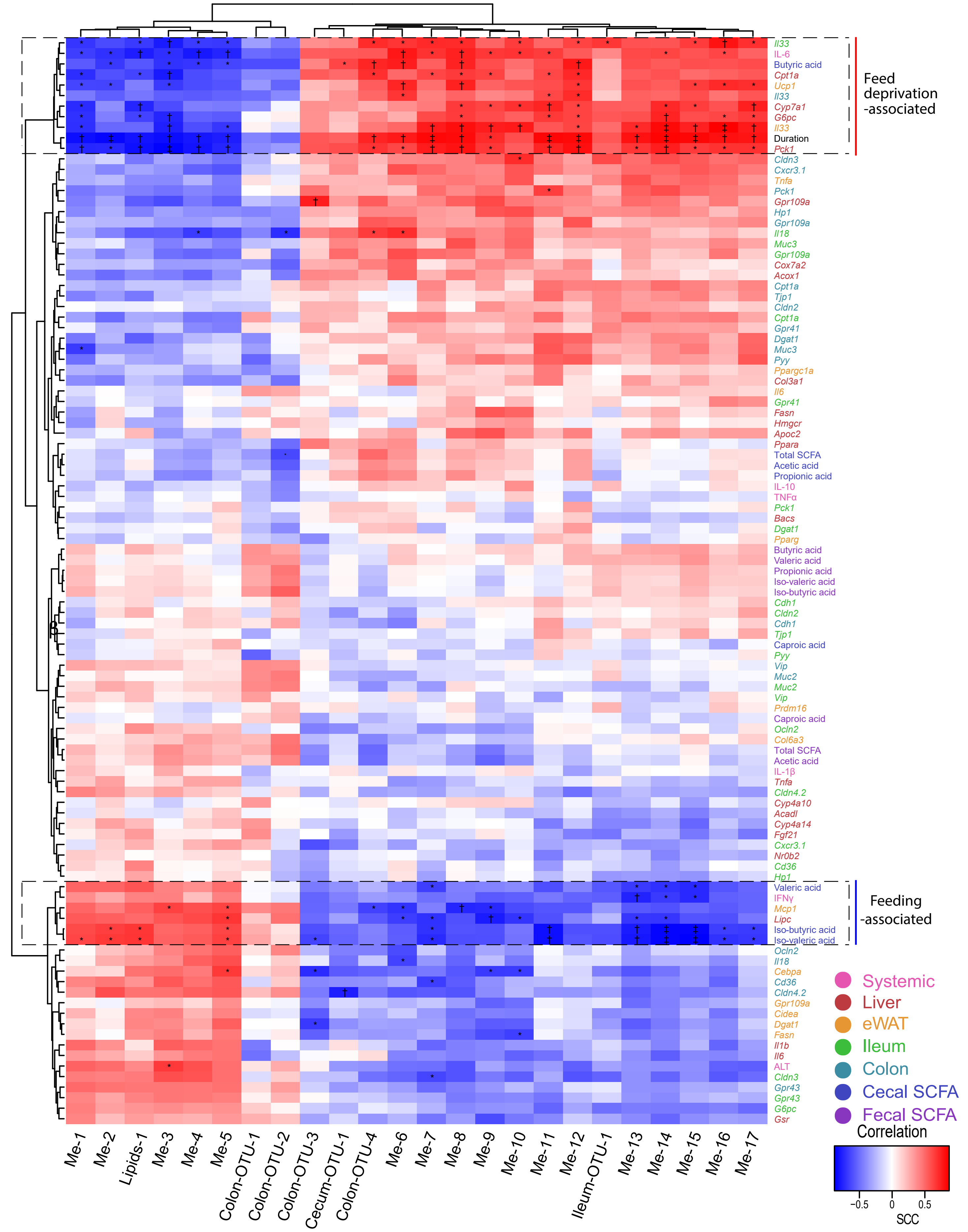

Figure S4. Integration of host parameters and bacterial fermentation products with the feed deprivation-dependent gut microbiota, liver lipid and urine metabolite clusters.
Correlation heatmap of hierarchically clustered, z-score-normalized host parameters (y-axis) against the eigengenes of feed deprivation-dependent lipid, metabolite and OTU clusters (x-axis). Duration of feed deprivation is included as a host parameter (8 hours and 16 minutes to 11 hours and 32 minutes for individual feed deprived mice). The coloring of host parameters corresponds to tissue origin as indicated in the legend. Heatmap color represents Spearman's rho-values (red, positive SCC; blue, negative SCC). Asterisks mark FDR-adjusted significant correlations (*, FDR < 0.05; †, FDR < 0.01; ‡, FDR < 0.001). The dotted line indicates the major host factors associated with duration of feed deprivation, as shown in figure 1.

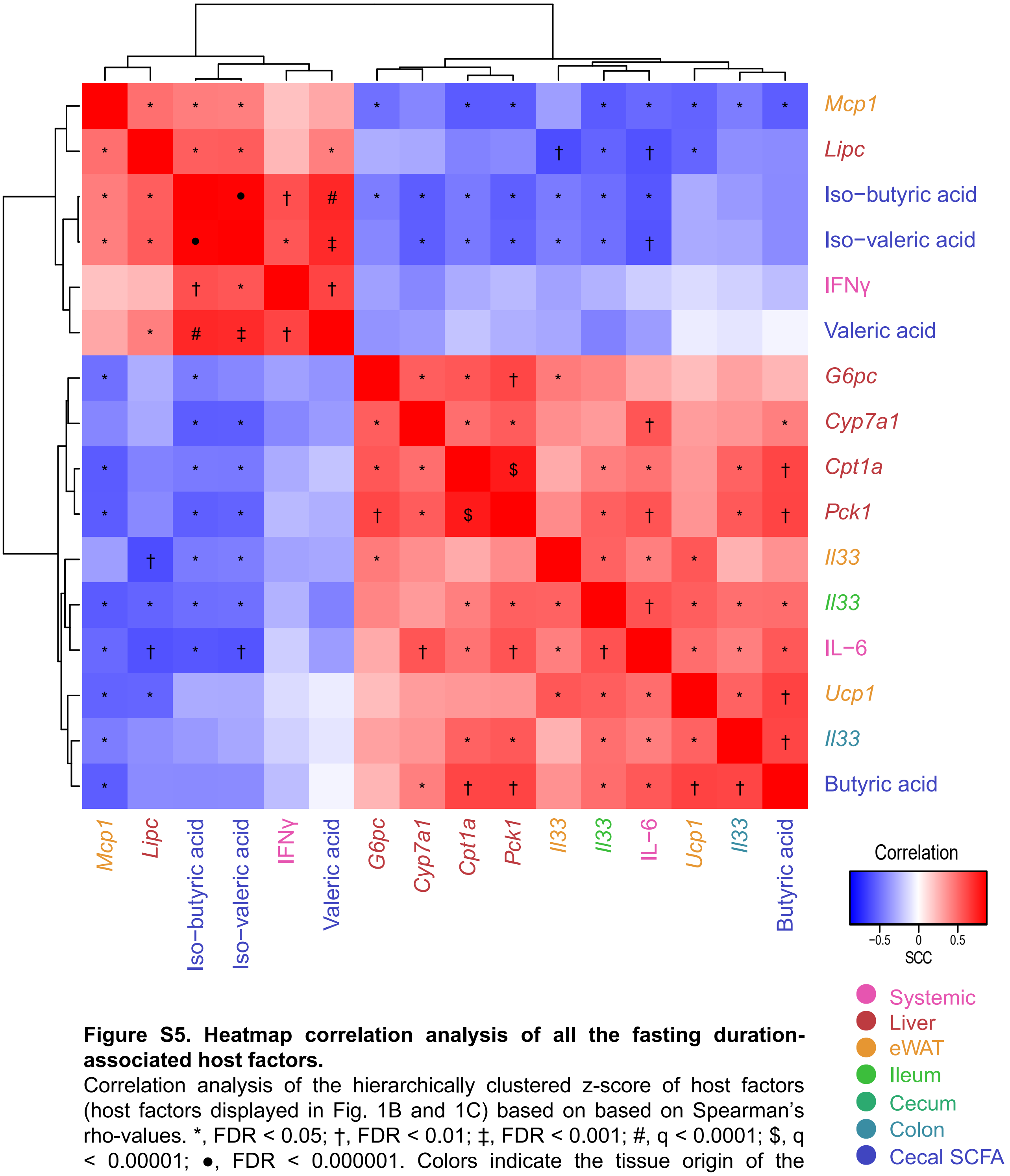

Figure S5. Heatmap correlation analysis of all the fasting duration-associated host factors.
Correlation analysis of the hierarchically clustered z-score of host factors (host factors displayed in Fig. 1B and 1C) based on based on Spearman's rho-values. *, FDR < 0.05; †, FDR < 0.01; ‡, FDR < 0.001; #, q < 0.0001; $, q < 0.00001; ●, FDR < 0.000001. Colors indicate the tissue origin of the individual variable (see colored legend).

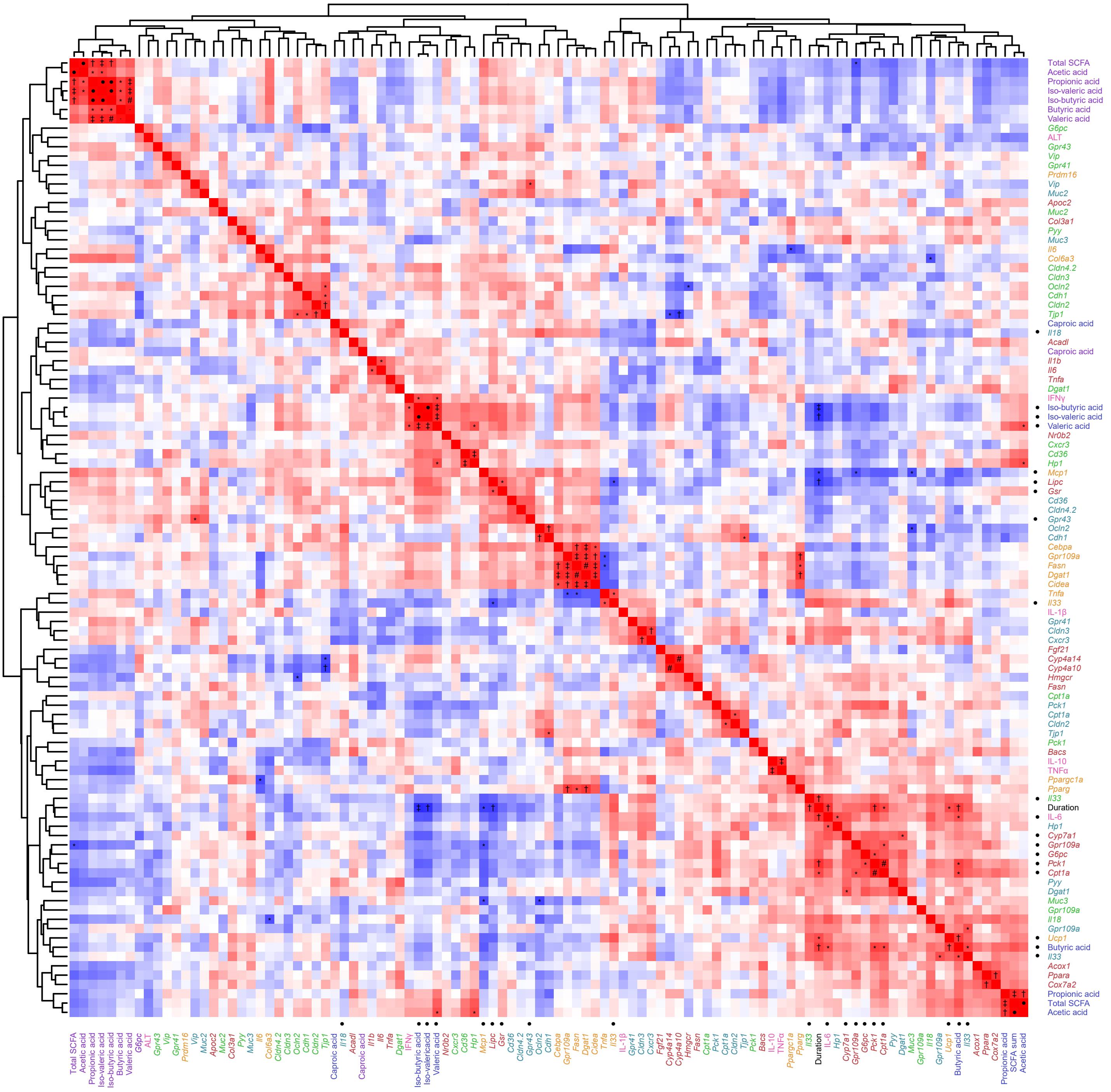

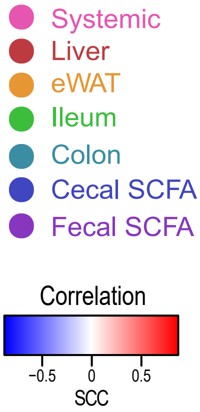

Figure S6. Heatmap correlation analysis of all host-related factors. Correlation analysis of the hierarchically clustered z-scores of host factors (colored boxed in Fig. 1A) based on Spearman's rho-values. Spearman's correlation; *, FDR < 0.05; †, FDR < 0.01; ‡, FDR < 0.001; #, q < 0.0001; $, q < 0.00001; ●, FDR < 0.000001. Host factors that are significantly different between fed and feed deprived mice are represented by a ●. The color of the nodes indicates the tissue origin of the individual parameters (see colored legend).

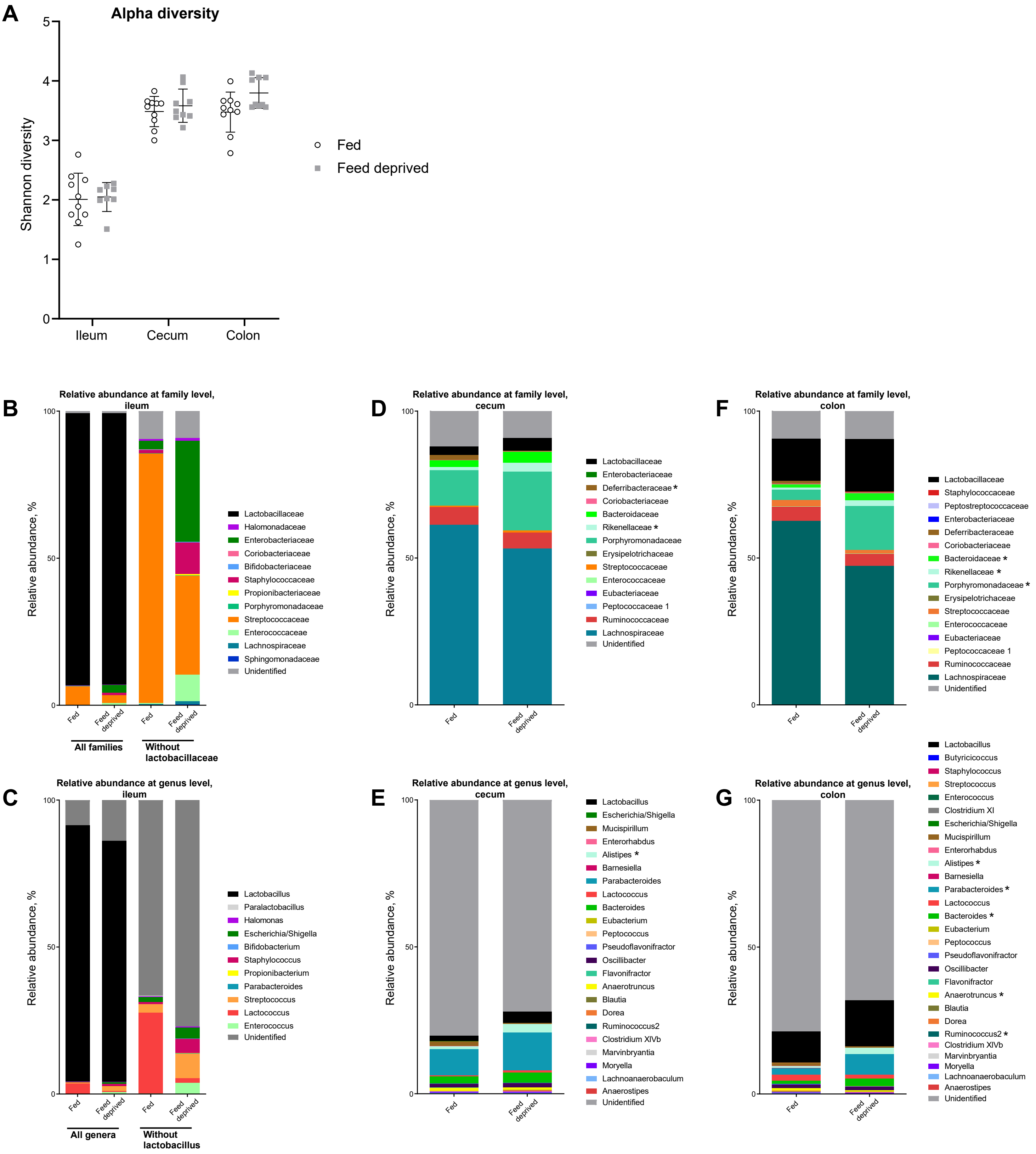

Figure S7. Relative abundance at family and genus level of the ileal, cecal and colonic microbiota. Samples from fed (n=10) and feed deprived (n=8 for ileum, n=9 for cecum and colon) obese mice were subjected to 16s rRNA gene amplicon sequencing. Ileal, cecal and colonic α-diversity of fed and feed deprived mice (A). Identified OTUs within ileum, cecum and colon were color-coded according to family (B, D, F), and genus (C, E, G). Statistical comparison of the two groups was done by permutation test (10,000 times). Asterisks represent fraction of times that permuted differences assessed by Welch's t-test were greater than or equal to real differences (*, FDR < 0.05).

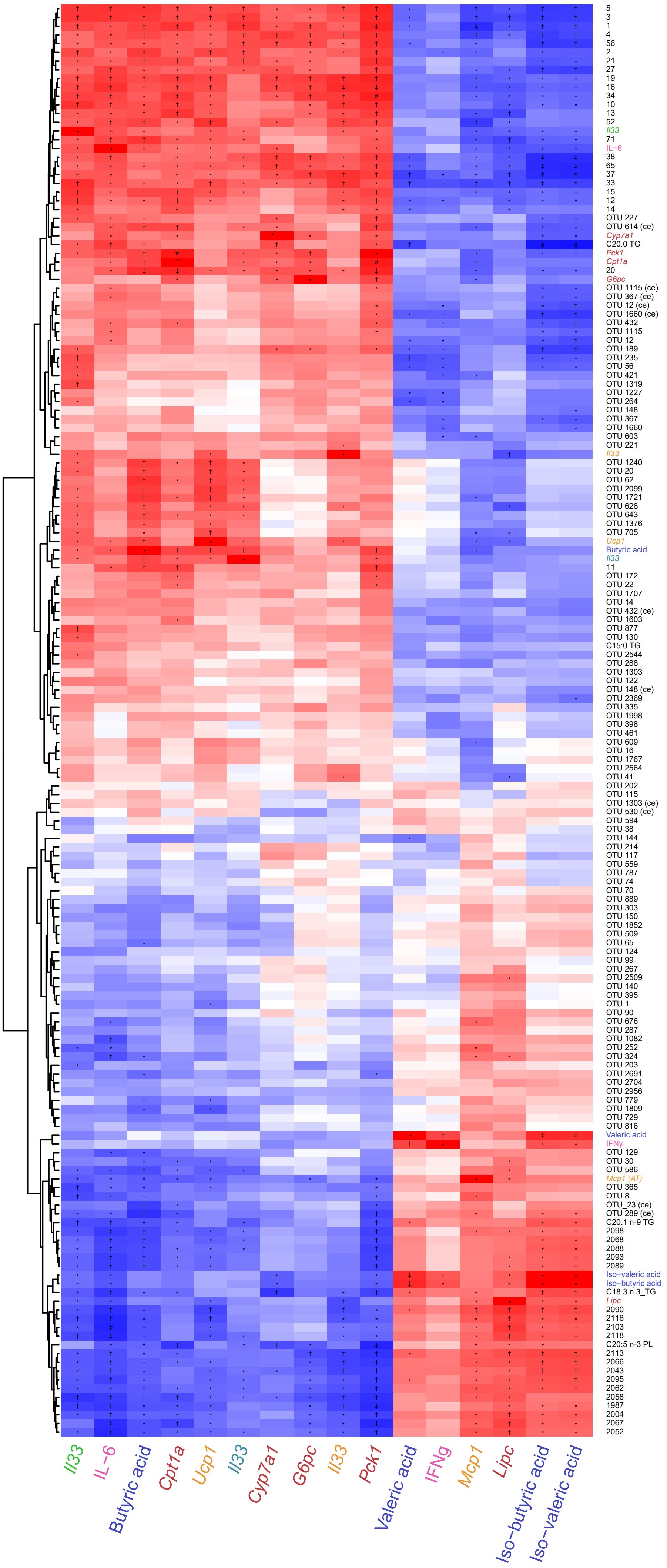

Figure S8. Heatmap correlation analysis of filtered factors associated with duration of feed deprivation.
Correlation analysis of hierarchically clustered, z-scored feed deprivation-dependent host factors (from Fig. 1B and 1C) versus the 43 metabolites, 100 OTUs and five liver lipids associated with duration of feed deprivation. Identifiers for the 43 metabolites, 100 OTUs and 5 lipids are shown in Table S2A-C. The heatmap color refers to Spearman's correlation coefficient (SCC); *, FDR < 0.05; †, FDR < 0.01; ‡, FDR < 0.001; #, q < 0.0001. Colors of host factors indicate the tissue origin of the individual variable (see colored legend).

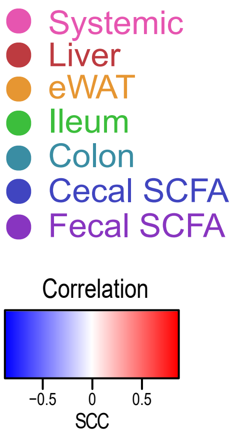

Table S1. RT-qPCR primer sequences.

Gene	Forward primer	Reverse primer	Probe
Ppara	TGCAACTTCTCAATGTAGCCT	AATGCCTTAGAACTGGATGACA	/56-FAM/AATTTGCTG/ZEN/TGGAGATCGGCCTGG/3IABkFQ/
Dgat1	CACCAGGATGCCATACTTGA	TCTTTGTTCAGCTCAGACAGTG	/56-FAM/AGCATCACC/ZEN/ACACACCAATTCAGGA/3IABkFQ/
Cd36	GTTCTGAAACATCTGGACTTGC	ACTGTACATCTTATGGTGTGCT	/56-FAM/AGGCTTTCC/ZEN/TTCTTTGCATTTGCCA/3IABkFQ/
Ffar2/Gpr43	CTGTCCTCAGTCCAAAGCTG	AGTGCTGGGATTACAGGTTC	/56-FAM/AGCCACCTG/ZEN/CCAGAACTCCTTG/3IABkFQ/
Ffar3/Gpr41	AAGCCAGTAATTGCCAAGAAAG	GCAGCAGAGTGCCAGTT	/56-FAM/CCCCATGGT/ZEN/CACAGATGCAGAGTAT/3IABkFQ/
Niacr1/Gpr109a	GAGTAGATGTCACAGTTGCGT	TCATTTGCTTCCTACCCAGTG	/56-FAM/AGAGAAGCC/ZEN/AGAAGATGCGGATGC/3IABkFQ/
Fgf21	GGGATGGGTCAGGTTCAGA	CAGCCTTAGTGTCTTCTCAGC	/56-FAM/TCAACACAG/ZEN/GAGAAACAGCCATTCACT/3IABkFQ/
Pyy	GTCGCTGTCGTCTGTGAAG	CGCCACTACCTCAACCTG	/56-FAM/CCCGCAGCT/ZEN/CTGTTCTCCAAACT/3IABkFQ/
Vip	GAGTATCAGGAATGCCAGGAA	GAACTTCAGCACCCTAGACAG	/56-FAM/ACCGAGATG/ZEN/GAAGCCAGAAGCAA/3IABkFQ/
Il1b	CTCTTGTTGATGTGCTGCTG	GACCTGTTCTTTGAAGTTGACG	/56-FAM/TTCCAAACC/ZEN/TTTGACCTGGGCTGT/3IABkFQ/
Il18	CACAGCCAGTCCTCTTACTTC	TCGTTGACAAAAGACAGCCT	/56-FAM/TGCCAGTGA/ZEN/ACCCCAGACCAG/3IABkFQ/
Il33	TCATGTTCACCATCAGCTTCT	GTGCTACTACGCTACTATGAGTC	/56-FAM/ACCGTCGCC/ZEN/TGATTGACTTGCA/3IABkFQ/
Ucp1	CACACCTCCAGTCATTAAGCC	CAAATCAGCTTTGCCTCACTC	/56-FAM/AAACACCTG/ZEN/CCTCTCTCGGAAACAA/3IABkFQ/
Prdm16	TCGCTCAATAGTCTTGTTCTCAA	AGAAGTCCCATACACAACCG	/56-FAM/TCGCAACAT/ZEN/GCTCAAGCCAAACC/3IABkFQ/
Cidea	GTAACCAGGCCAGTTGTGAT	TCAAACCATGACCGAAGTAGC	/56-FAM/AGACATCCA/ZEN/GAGTCTTGCTGATAAGTTCCT/3IABkFQ/
Ppargc1a	TCGCTCAATAGTCTTGTTCTCAA	AGAAGTCCCATACACAACCG	/56-FAM/TCGCAACAT/ZEN/GCTCAAGCCAAACC/3IABkFQ/

Forward, reverse and probe sequences for validated, predesigned TaqMan primer-probe pairs not included in Zhang et al, 2017[11].

Table S2. Identifiers for the duration of feed deprivation-dependent urine metabolites, intestinal OTUs and liver lipids.

A) The 100 OTUs from any of the WGCNA clusters Ileum-OTU-1, Cecum-OTU-1 or Colon-OTU-1 to -4. The lowest taxonomical identification is noted in “Taxonomy”.

OTU ID	Tissue	Taxonomy‡	Cluster
OTU 1	Colon	*Lachnospiraceae*	Colon-OTU-2
OTU 8	Colon	*Lachnospiraceae*	Colon-OTU-4
OTU 12 (ce)	Colon	*Alistipes*	Cecum-OTU-1
OTU 12	Cecum	*Alistipes*	Colon-OTU-3
OTU 14	Colon	*Porphyromonadaceae*	Colon-OTU-4
OTU 16	Colon	*Peptococcus*	Colon-OTU-4
OTU 20	Colon	*Porphyromonadaceae*	Colon-OTU-4
OTU 22	Colon	*Bacteroides*	Colon-OTU-3
OTU 23 (ce)	Cecum	*Lachnospiraceae*	Cecum-OTU-1
OTU 30	Colon	*Anaerotruncus*	Colon-OTU-2
OTU 38	Colon	*Clostridiales*	Colon-OTU-2
OTU 41	Colon	*Oscillibacter*	Colon-OTU-4
OTU 56	Ileum	*Staphylococcus*	Ileum-OTU-1
OTU 62	Colon	*Porphyromonadaceae*	Colon-OTU-4
OTU 65	Colon	*Mucispirillum*	Colon-OTU-1
OTU 70	Colon	*Firmicutes*	Colon-OTU-1
OTU 74	Colon	*Lachnospiraceae*	Colon-OTU-2
OTU 90	Colon	*Butyricicoccus*	Colon-OTU-4
OTU 99	Colon	*Coriobacteriaceae*	Colon-OTU-2
OTU 115	Colon	*Enterococcus*	Colon-OTU-4
OTU 117	Colon	*Lachnospiraceae*	Colon-OTU-2
OTU 122	Colon	*Firmicutes*	Colon-OTU-4
OTU 124	Colon	*Ruminococcaceae*	Colon-OTU-1
OTU 129	Colon	*Lachnospiraceae*	Colon-OTU-2
OTU 130	Colon	*Porphyromonadaceae*	Colon-OTU-4
OTU 140	Colon	*Clostridiales*	Colon-OTU-2
OTU 144	Colon	*Lachnoanaerobaculum*	Colon-OTU-1
OTU 148 (ce)	Cecum	*Alistipes*	Cecum-OTU-1
OTU 148	Colon	*Alistipes*	Colon-OTU-3
OTU 150	Colon	*Mucispirillum*	Colon-OTU-1
OTU 172	Colon	*Bacteroides*	Colon-OTU-3
OTU 189	Ileum	*Enterococcus*	Ileum-OTU-1
OTU 202	Colon	*Escherichia/Shigella*	Colon-OTU-4
OTU 203	Colon	*Ruminococcus2*	Colon-OTU-4
OTU 214	Colon	*Lachnospiraceae*	Colon-OTU-2
OTU 221	Colon	*Clostridiales*	Colon-OTU-4
OTU 227	Ileum	*Enterococcus*	Ileum-OTU-1
OTU 235	Ileum	*Staphylococcus*	Ileum-OTU-1
OTU 252	Colon	*Lachnospiraceae*	Colon-OTU-2
OTU 264	Ileum	*Staphylococcus*	Ileum-OTU-1
OTU 267	Colon	*Lachnospiraceae*	Colon-OTU-2
OTU 287	Colon	*Lachnospiraceae*	Colon-OTU-2
OTU 288	Colon	*Clostridium XI*	Colon-OTU-4
OTU 289 (ce)	Cecum	*Lachnospiraceae*	Cecum-OTU-1
OTU 303	Colon	*Mucispirillum*	Colon-OTU-1
OTU 324	Colon	*Lachnospiraceae*	Colon-OTU-2
OTU 335	Ileum	*Lactococcus*	Ileum-OTU-1
OTU 365	Colon	*Lachnospiraceae*	Colon-OTU-4
OTU 367 (ce)	Cecum	*Alistipes*	Cecum-OTU-1
OTU 367	Colon	*Alistipes*	Colon-OTU-3
OTU 395	Colon	*Clostridiales*	Colon-OTU-2
OTU 398	Colon	*Bacteria*	Colon-OTU-3
OTU 421	Ileum	*Enterococcus*	Ileum-OTU-1
OTU 432 (ce)	Cecum	*Alistipes*	Cecum-OTU-1
OTU 432	Colon	*Alistipes*	Colon-OTU-3
OTU 461	Colon	*Bacteria*	Colon-OTU-3
OTU 509	Colon	*Mucispirillum*	Colon-OTU-1
OTU 530 (ce)	Cecum	*Parabacteroides*	Cecum-OTU-1
OTU 559	Colon	*Lachnospiraceae*	Colon-OTU-4
OTU 586	Colon	*Lachnospiraceae*	Colon-OTU-2
OTU 594	Colon	*Clostridiales*	Colon-OTU-2
OTU 603	Colon	*Bacteria*	Colon-OTU-4
OTU 609	Colon	*Peptococcus*	Colon-OTU-4

OTU 614 (ce)	Cecum	*Alistipes*	Cecum-OTU-1
OTU 628	Colon	*Bacteroidetes*	Colon-OTU-4
OTU 643	Colon	*Bacteroidetes*	Colon-OTU-4
OTU 676	Colon	*Lachnospiraceae*	Colon-OTU-2
OTU 705	Colon	*Bacteroidetes*	Colon-OTU-4
OTU 729	Colon	*Clostridiales*	Colon-OTU-2
OTU 779	Colon	*Lachnospiraceae*	Colon-OTU-1
OTU 787	Colon	*Clostridiales*	Colon-OTU-2
OTU 816	Colon	*Lachnospiraceae*	Colon-OTU-2
OTU 877	Colon	*Bacteroidales*	Colon-OTU-4
OTU 889	Colon	*Anaerotruncus*	Colon-OTU-2
OTU 1082	Colon	*Lachnospiraceae*	Colon-OTU-2
OTU 1115 (ce)	Cecum	*Alistipes*	Cecum-OTU-1
OTU 1115	Colon	*Alistipes*	Colon-OTU-3
OTU 1227	Ileum	*Staphylococcus*	Ileum-OTU-1
OTU 1240	Colon	*Porphyromonadaceae*	Colon-OTU-4
OTU 1303 (ce)	Cecum	*Parabacteroides*	Cecum-OTU-1
OTU 1303	Colon	*Parabacteroides*	Colon-OTU-3
OTU 1319	Ileum	*Staphylococcus*	Ileum-OTU-1
OTU 1376	Colon	*Porphyromonadaceae*	Colon-OTU-4
OTU 1603	Colon	*Clostridiales*	Colon-OTU-3
OTU 1660 (ce)	Cecum	*Alistipes*	Cecum-OTU-1
OTU 1660	Colon	*Alistipes*	Colon-OTU-3
OTU 1707	Colon	*Bacteroides*	Colon-OTU-3
OTU 1721	Colon	*Porphyromonadaceae*	Colon-OTU-4
OTU 1767	Colon	*Peptococcus*	Colon-OTU-4
OTU 1809	Colon	*Lachnospiraceae*	Colon-OTU-2
OTU 1852	Colon	*Mucispirillum*	Colon-OTU-1
OTU 1998	Colon	*Bacteria*	Colon-OTU-4
OTU 2099	Colon	*Bacteroidetes*	Colon-OTU-4
OTU 2369	Colon	*Porphyromonadaceae*	Colon-OTU-4
OTU 2509	Colon	*Oscillibacter*	Colon-OTU-2
OTU 2544	Colon	*Porphyromonadaceae*	Colon-OTU-4
OTU 2564	Colon	*Oscillibacter*	Colon-OTU-4
OTU 2691	Colon	*Lachnospiraceae*	Colon-OTU-4
OTU 2704	Colon	*Lachnospiraceae*	Colon-OTU-2
OTU 2956	Colon	*Lachnospiraceae*	Colon-OTU-4

‡ The lowest identified taxonomic level is shown, if classification at family-level was not possible.

Table S2. Identifiers for the duration of feed deprivation-dependent urine metabolites, intestinal OTUs and liver lipids.

ID	Name	*m/z* ratio	rt (sec)	Ion	Database	ID level	Log2 fold change	Cluster
16	Unknown	544.9673	37			4	2.52	Me-3
19	Unknown	321.9758	37			4	2.25	Me-3
34	Unknown	446.9905	37			4	2.13	Me-3
52	Unknown	196.9611	38			4	2.08	Me-8
37	Unknown	540.0867	39			4	2.3	Me-5
2004	Disaccharide	381.0793	42	[M+K]	HMDB	3	-2.06	Me-5
2103	Dipeptide - Prolyl-glutamine	244.1291	42	[M+H]	HMDB	4	-2.95	Me-6
2066	Disaccharide	360.1500	42	[M+NH4]	HMDB	4	-3.01	Me-5
2062	Disaccharide (isotope of 2066)	361.1531	43	[M+NH4]	HMDB	4	-2.83	Me-5
2113	Disaccharide fragment	307.1019	43	[M+H]	HMDB	4	-3.6	Me-5
2043	Disaccharide fragment	289.0919	43	[M+H]	HMDB	4	-2.46	Me-5
2118	Dipeptide fragment	217.1174	43	[M+H]	METLIN	4	-6.18	Me-6
21	Unknown	159.0287	49			4	2.67	Me-11
71	Gluconolactone, Galactonolactone *or* Gulonolactone fragment	161.0444	65	[M+H-H2O]	HMDB	3	2.04	Me-6
33	Gluconolactone, Galactonolactone *or* Gulonolactone	196.0813	66	[M+NH4]	HMDB	3	2.26	Me-6
2	Unknown	226.1284	78			4	8.26	Me-6
4	Unknown	328.1231	80			4	5.19	Me-11
2058	Hydroxybutyrylcarnitine	248.1490	82	[M+H]	HMDB	3	-2.03	Me-3
1987	Unknown	297.1443	122			4	-2.06	Me-4
2090	Unknown	255.1634	161			4	-2.83	Me-6
2116	Dipeptide fragment	254.1609	161	[M+H-H2O] or [M+NH4-H2O]	HMDB	4	-4.96	Me-6
56	Unknown	266.1054	177			4	2.28	Me-11
11	Unknown	516.7704	211			4	2.58	Me-4
65	Unknown	232.1000	259			4	2.06	Me-11
27	Unknown (isotope of 38)	271.0800	262			4	2.16	Me-11
38	Unknown	270.0771	262			4	2.21	Me-11
5	Unknown	524.1183	291			4	4.82	Me-6
20	Unknown	473.2967	301			4	2.53	Me-11
2067	Unknown	307.1174	313			4	-2.28	Me-4
2052	Unknown	360.1652	313			4	-2.1	Me-4
10	Tetrahydrocortisone isotope *or* Dihydrocortisol isotope	366.2352	314	[M+H]	HMDB	3	2.78	Me-1
15	Tetrahydrocortisone *or* Dihydrocortisol	365.2320	314	[M+H]	HMDB	3	2.72	Me-1
2095	Unknown	490.1740	318			4	-2.75	Me-4
13	18-Hydroxycortisol *or* 6-beta-hydrocortisol	379.2113	319	[M+H]	HMDB	3	2.84	Me-3
1	Hydroxysanguinarine	348.0866	340	[M+H]	METLIN	3	9.74	Me-6
3	Hydroxysanguinarine isotope	349.0898	341	[M+H]	METLIN	3	7.32	Me-6
2068	O-glycosyl compound fragment (isotope of 2093)	375.1841	351	[M+NH4]	HMDB	4	-2.98	Me-4
2088	O-glycosyl compound fragment	379.1362	351	[M+Na]	HMDB	4	-2.86	Me-4
2098	O-glycosyl compound fragment	417.1867	351	[M+H]	HMDB	4	-3.39	Me-4
2093	O-glycosyl compound fragment	374.1809	351	[M+NH4]	HMDB	4	-3.28	Me-4
2089	O-glycosyl compound fragment	321.1331	351	[M+H]	HMDB	4	-3.38	Me-4
14	Cortisol *or* 18-Hydroxycorticosterone	363.2166	374	[M+H]	HMDB	3	2.53	Me-1
12	Cortisol isotope *or* 18-Hydroxycorticosterone isotope	364.2200	374	[M+H]	HMDB	3	2.68	Me-1

B) The 43 metabolites putatively annotated using m/z ratios using databases from either METLIN or HMDB. Metabolites that could not be annotated are named “Unknown / noise” and for some metabolites the database search resulted in several likely candidates.

Table S2. Identifiers for the duration of feed deprivation-dependent urine metabolites, intestinal OTUs and liver lipids.

C) The five liver lipids from the Lipids-1 cluster.

ID	Type	Cluster
C20:5 *n*-3	Phospholipid	Lipids-1
C18:3 *n*-3	Triglyceride	Lipids-1
C20:1 *n*-9	Triglyceride	Lipids-1
C20:0	Triglyceride	Lipids-1
C15.0	Triglyceride	Lipids-1

Table S3. *But* and *Buk* enzymes in bacterial families.

Family	Total taxIDs	taxIDs with hits	TaxIDs with BUK pathway	TaxIDs with BUT pathway	TaxIDs with either BUT or BUK pathway	% BUK of total	% BUT of total	% BUT/BUK of total	% BUK of hits	% BUT of hits	% BUT/BUK of hits
Bacteroidaceae	882	132	79	0	79	9.0%	0.0%	9.0%	59.8%	0.0%	59.8%
Coriobacteriaceae	125	23	11	0	11	8.8%	0.0%	8.8%	47.8%	0.0%	47.8%
Deferribacteraceae	76	6	4	0	4	5.3%	0.0%	5.3%	66.7%	0.0%	66.7%
Enterobacteriaceae	19011	512	196	0	196	1.0%	0.0%	1.0%	38.3%	0.0%	38.3%
Enterococcaceae	2756	75	52	0	52	1.9%	0.0%	1.9%	69.3%	0.0%	69.3%
Lachnospiraceae	1463	286	175	80	201	12.0%	5.5%	13.7%	61.2%	28.0%	70.3%
Peptococcaceae	597	72	43	3	43	7.2%	0.5%	7.2%	59.7%	4.2%	59.7%
Peptostreptococcaceae	651	89	41	5	42	6.3%	0.8%	6.5%	46.1%	5.6%	47.2%
Porphyromonadaceae	448	52	26	0	26	5.8%	0.0%	5.8%	50.0%	0.0%	50.0%
Rikenellaceae	107	31	24	0	24	22.4%	0.0%	22.4%	77.4%	0.0%	77.4%
Ruminococcaceae	741	110	59	12	65	8.0%	1.6%	8.8%	53.6%	10.9%	59.1%
Staphylococcaceae	8884	117	60	0	60	0.7%	0.0%	0.7%	51.3%	0.0%	51.3%
Streptococcaceae	4246	215	87	0	87	2.0%	0.0%	2.0%	40.5%	0.0%	40.5%

The presence or absence of enzymes of the butyrate kinase (*Buk*) or butyrate transferase (*But*) pathway of bacterial families to which the OTUs in the clusters associated with feed deprivation belong.
